# High Efficacy but Low Potency of δ-Opioid Receptor-G Protein Coupling in Brij-58-Treated, Low-Density Plasma Membrane Fragments

**DOI:** 10.1371/journal.pone.0135664

**Published:** 2015-08-18

**Authors:** Lenka Roubalova, Miroslava Vosahlikova, Jana Brejchova, Jan Sykora, Vladimir Rudajev, Petr Svoboda

**Affiliations:** 1 Department of Biomathematics, Institute of Physiology of the Czech Academy of Sciences, Prague, Czech Republic; 2 Department of Biophysical Chemistry, J. Heyrovsky Institute of Physical Chemistry of the Czech Academy of Sciences, Prague, Czech Republic; 3 Department of Neurochemistry, Institute of Physiology of the Czech Academy of Sciences, Prague, Czech Republic; Medical School of Hannover, GERMANY

## Abstract

**Principal Findings:**

HEK293 cells stably expressing PTX-insensitive δ-opioid receptor-Gi1α (C^351^I) fusion protein were homogenized, treated with low concentrations of non-ionic detergent Brij-58 at 0°C and fractionated by flotation in sucrose density gradient. In optimum range of detergent concentrations (0.025–0.05% w/v), Brij-58-treated, low-density membranes exhibited 2-3-fold higher efficacy of DADLE-stimulated, high-affinity [^32^P]GTPase and [^35^S]GTPγS binding than membranes of the same density prepared in the absence of detergent. The potency of agonist DADLE response was significantly decreased. At high detergent concentrations (>0.1%), the functional coupling between δ-opioid receptors and G proteins was completely diminished. The same detergent effects were measured in plasma membranes isolated from PTX-treated cells. Therefore, the effect of Brij-58 on δ-opioid receptor-G protein coupling was not restricted to the covalently bound G_i_1α within δ-opioid receptor-G_i_1α fusion protein, but it was also valid for PTX-sensitive G proteins of G_i_/G_o_ family endogenously expressed in HEK293 cells. Characterization of the direct effect of Brij-58 on the hydrophobic interior of isolated plasma membranes by steady-state anisotropy of diphenylhexatriene (DPH) fluorescence indicated a marked increase of membrane fluidity. The time-resolved analysis of decay of DPH fluorescence by the “wobble in cone” model of DPH motion in the membrane indicated that the exposure to the increasing concentrations of Brij-58 led to a decreased order and higher motional freedom of the dye.

**Summary:**

Limited perturbation of plasma membrane integrity by low concentrations of non-ionic detergent Brij-58 results in alteration of δ-OR-G protein coupling. Maximum G protein-response to agonist stimulation (efficacy) is increased; affinity of response (potency) is decreased. The total degradation plasma membrane structure at high detergent concentrations results in diminution of functional coupling between δ-opioid receptors and G proteins.

## Introduction

Membrane domains/caveolae are plasma membrane (PM) compartments enriched in cholesterol and glycosphingolipids, which are resistant to solubilization by non-ionic detergents such as Triton X-100 at 0°C [1–17]. Besides the resistance to detergent solubilization, the second characteristic feature of these PM structures is the low buoyant density when exposed to the high centrifugation force. They migrate up to the low-density area equivalent in density to 15–20% w/v sucrose. In this way, detergent-resistant membrane domains (DRMs) are separated from the bulk of plasma membranes recovered in 35% w/v sucrose. Other cell constituents remain either in “cushion solution”, i.e. in the layer of highest density (40–45% w/v sucrose) or sediment down to gradient pellet (nuclear fragments).

The prototypical detergent used for preparation of DRMs is Triton X-100. The cell homogenate or membrane preparations are treated with Triton X-100 for 60 min at 0–4°C and fractionated by flotation in density gradients [7,8,18]. Unfortunately, these procedures using the high detergent concentrations (0.5–1%), result in a loss of functional coupling between GPCR and their cognate signaling molecules, trimeric G proteins and adenylyl cyclase (AC) [19]. Membrane domains/caveolae were also prepared by detergent-free method using 1M Na_2_CO_3_ (pH 11) and sonication [20,21], however, due to the highly alkaline pH of Na_2_CO_3_, the responsiveness of G proteins and AC activity to GPCR agonist stimulation was also diminished [19,22]. Isolation of the highly purified caveolae from human fibroblasts by detergent-free and alkaline-free procedure was reported by Smart et al. [23], but the amount of protein (10 μg) recovered in final preparation represented 0.13–0.14% of total protein originally present in cell homogenate (7–8 mg). Obviously, the low amount of protein in final preparation of membrane domains/caveolae is not sufficient for characterization of GPCR by radioligand binding assays using ^3^H-labelled ligands (40–60 Ci/mmol) and measurements of G protein response to agonist stimulation by high-affinity [^32^P]GTPase or [^35^S]GTPγS binding assays.

Therefore, we tried to improve the methodological conditions for isolation of DRMs and tested different detergents at varying concentrations and detergent/protein ratios with the aim to prepare membrane domains with preserved functional coupling between -opioid receptors (δ-OR) and G proteins. We also tried to find some reasonable compromise between purity and amount of protein recovered in final preparation of DRMs. When using Brij-58 at low concentrations and in optimum range of protein amount in cell homogenate (10–15 mg × ml^-1^), detergent-treated membranes floated up to the low-density end of sucrose gradient and were functional in terms of receptor-G protein coupling. Surprisingly, the efficacy of δ-OR when increasing the high-affinity [^32^P]GTPase activity or [^35^S]GTPγS binding was 2-3-fold higher than in membranes prepared in the absence of detergent. The potency of G protein response to agonist stimulation, however, was decreased.

## Materials and Methods

### Chemicals

δ-OR agonist [^3^H]DADLE [Enkephalin (2-D-Alanine-5-D-Leucine), 50 Ci/mmol, NET 648] was purchased from PerkinElmer (Boston, MA, USA); δ-OR antagonist [^3^H]naltrindole (60 mCi/mmol, ART0549) was from ARC (St. Louis, MO, USA). Guanosine-5´-[γ-^35^S]thiotriphosphate ([^35^S]GTPγS; 1115 Ci/mmol, NEG030H) was from PerkinElmer (Boston, MA, USA); guanosine-5´-[γ-^32^P] triophosphate ([γ^32^P]GTP; 30 Ci/mmol, 35007) was from MP Biomedicals (Santa Ana, CA, USA). Complete protease inhibitor cocktail was from Roche Diagnostic (cat. no. 1697498, Mannheim, Germany), geneticin and hygromycin were from Life Technologies-Gibco (Paisley, Scotland). All other chemicals and drugs were purchased from Sigma-Aldrich.

### Cell culture

HEK293T cells stably expressing δ-OR-G_i_1α (C^351^I) fusion protein were cultivated in Dulbecco’s modified Eagle’s medium (Sigma) supplemented with 2 mM (0.292 g × l^-1^) L-glutamine and 10% v/v newborn calf serum at 37°C as described previously [24]. Geneticin (800 μg) or hygromycin (200 μg) were included in the course of cell cultivation. The cells were grown to 80% confluency before harvesting and the beginning of experiments. When indicated, the cells were incubated with (+PTX) pertussis toxin (10 ng × ml^-1^) for 24 h before harvesting.

### Preparation of post-nuclear fraction

δ-OR-G_i_1α (C^351^I)-HEK293 cells (δ-OR-G_i_1α cells for short) were harvested from 15 flasks (80 cm^2^) by centrifugation for 10 min at 300 × g (0–4°C) and homogenized in 50 mM Tris-HCl, pH 7.6, 3 mM MgCl_2_, 1 mM EDTA (TME buffer) containing 1 mM fresh PMSF plus Complete protease inhibitors cocktail in Teflon–glass homogenizer for 7 min at 1360 rpm on ice. The cell homogenate was centrifuged for 7 min at 3500 rpm (1160 × g) to separate cell debris and nuclear fraction (remaining in the sediment) from post-nuclear supernatant, PNS.

### Isolation of low-density membranes (LDM) in the presence of low concentrations of non-ionic detergent Brij-58

δ-OR-G_i_1α (C^351^I)-HEK293 cells were washed in PBS, harvested from totally 60 flasks (80 cm^2^) by centrifugation for 10 min at 300 × g (0–4°C). Homogenization was performed in TME buffer containing 1 mM fresh PMSF plus Complete protease inhibitors cocktail in Teflon–glass homogenizer for 7 min at 1360 rpm on ice. The cell homogenate was divided into four identical portions having exactly the same volume. The first portion was diluted with 1/10 volume of water (no detergent). The second, third and fourth portion was diluted with 1/10 volume of water plus 10% w/v Brij-58 to a final concentration of 0.025, 0.05 and 0.1% w/v Brij-58, respectively. Exactly 2 ml volumes of these four samples were transferred into Beckman SW41 tube, mixed with 2 ml of 80% w/v sucrose, overlaid with 35%, 30%, 25%, 20% and 15% w/v sucrose (1.5 ml each) and centrifuged for 24 hours at 187,000 × g (0–4°C). Centrifugation resulted in the separation of two clearly visible layers. The lower, optically dense layer represented the plasma membrane fraction (PM). The low-density membranes (LDM) were visible as a hazy area in the upper part of sucrose gradient. Sucrose fractions 1–12 (1 ml) were collected from top to bottom of the centrifuge tube, snap frozen in liquid nitrogen and stored at -80°C.

### Isolation of PM fraction in Percoll density gradient

δ-OR-G_i_1α (C^351^I)-HEK293 cells were cultivated in 60 flasks (80 cm^2^), washed in PBS and harvested by centrifugation for 10 min at 1800 rpm (300 × g) in 50 ml conical vials. The cell sediment was homogenized in 250 mM sucrose, 20 mM Tris-HCl, 3 mM MgCl_2_, 1 mM EDTA, pH 7.6 (STEM medium) plus fresh 1 mM PMSF and protease inhibitor cocktail in loosely-fitting, Teflon-glass homogenizer for 7 min at 1360 rpm on ice. The cell homogenate was centrifuged for 7 min at 3500 rpm (1160 × g) to separate cell debris and nuclear fraction (remaining in the sediment) from post-nuclear supernatant, PNS. A 3 ml volume of PNS was applied on the top of 20 ml of 30% v/v Percoll in thick polycarbonate Beckman Ti70 tubes. Centrifugation for 35 min at 30,000 rpm (66,200 × g) resulted in separation of two clearly visible layers. The upper layer represented the plasma membrane-enriched fraction (PM), the lower layer represented mitochondria. The upper layer was diluted 1:4 in STEM medium and centrifuged in Beckman Ti70 rotor for 1.5 hours at 50,000 rpm (175,000 × g). The PM sediment was removed from the compact, gel-like sediment of Percoll, re-homogenized by hand in a small volume of 50 mM Tris-HCl, 1 mM EDTA, pH 7.4 (TE buffer), frozen in liquid nitrogen and stored at -80°C.

### [^32^P]GTPase activity

When screening the density gradient profiles, constant volume aliquots (30 μl) of sucrose gradient fractions were incubated with or without 100 μM DADLE in reaction mix containing in a final volume of 100 μl 10 mM creatine phosphate, 5 units of creatine kinase, 0.5 μM GTP, 1 mM ATP, 0.1 mM adenosine-5´-O-(3-imidotriphosphate (App(NH)p), 1 mM ouabain, 100 mM NaCl, 5 mM MgCl_2_, 2 mM dithiothreitol, 0.1 mM EDTA, 40 mM Tris-HCl, pH 7.5 and [γ-^32^P]GTP (about 200,000 dpm per assay) for 30 min at 37°C. The enzyme reaction was terminated by transferring the reaction mix into the ice-cold water bath and adding 0.9 ml of 5% w/v charcoal in 10 mM phosphoric acid. After 5 min at 0°C, the charcoal was removed by centrifugation for 10 min at 10,000 × g and 0.5 ml of clear supernatant was used for determination of radioactivity by liquid scintillation.

Determination of V_max_ (GTP) and K_m_ (GTP) values of basal and DADLE (100 μM)-stimulated, high-affinity [^32^P]GTPase was performed by substrate (GTP) saturation assay as described before [24,25]. [^32^P]GTPase activity was measured as function of increasing GTP concentrations and the data expressed as Eadie-Hofstee plots. The V_max_ and K_m_ values were calculated by GraphPad Prism4.

Dose-response curves of agonist-stimulated [^32^P]GTPase were measured in a 10^−10^–10^−3^ M range of DADLE concentrations. Maximum DADLE-stimulated enzyme activity [V_max_ (DADLE)] and concentration inducing the half-maximum response [EC_50_ (DADLE)] were calculated by fitting the data by GraphPad Prism4. In all types of measurements of [^32^P]GTPase activity, the non-specific, low-affinity [^32^P]GTPase activity was measured in parallel assays containing 100 μM GTP and subtracted from the basal and DADLE-stimulated enzyme activity.

### [^35^S]GTPγS binding

Constant volume aliquots (20 μl) of gradient fractions were incubated in the absence (basal) or presence (agonist-stimulated) 100 μM DADLE in a final volume of 100 μl of reaction mix containing 20 mM HEPES, pH 7.4, 3 mM MgCl_2_, 100 mM NaCl, 2 μM GDP, 0.2 mM ascorbate and [^35^S]GTPγS (about 200,000 dpm per assay, ≈ 2nM) for 30 min at 30°C. The binding reaction was discontinued by dilution with 3 ml of ice-cold 20 mM HEPES, pH 7.4, 3 mM MgCl_2_ and filtration through Whatman GF/C filters in Brandel cell harvester. Radioactivity remaining on the filters was determined by liquid scintillation using Rotiszint eco plus cocktail. Non-specific [^35^S]GTPγS binding was determined in parallel assays containing 10 μM unlabeled GTPγS.

Dose-response curves of DADLE-stimulated [^35^S]GTPγS binding were measured in a 10^−10^–10^−3^ M range of DADLE concentrations and maximum response and EC_50_ values calculated by GraphPad Prism4. In competitive [^35^S]GTPγS/GTPγS binding experiments, [^35^S]GTPγS binding ± 100 μM DADLE was measured in the presence of increasing GTPγS concentrations and B_0_ (GTPγS) and IC_50_ (GTPγS) values were determined by GraphPad Prism4.

### [^3^H]naltrindole binding

Post-nuclear supernatant (PNS), LDM and 0.025% Brij-58-treated LDM were incubated in TME buffer with increasing concentrations of [^3^H]naltrindole (0.1–10.8 nM). Non-specific binding was determined in the presence of 100 μM naloxone. Binding reaction was continued for 60 min at 30°C, the bound and free radioactivity separated by filtration through GF/B filters in Brandel cell harvestor, the filters were washed three times with 3 ml ice-cold TME and the bound radioactivity was determined by liquid scintillation. Binding data were fitted with the rectangular hyperbola and K_d_ and B_max_ values calculated by GraphPad Prism4.

### [^3^H]DADLE binding

LDM, 0.025% Brij-58-treated LDM or Percoll-purified PM were incubated in TME buffer with increasing concentrations of [^3^H]DADLE (0.2–39.3 nM) for 60 min at 30°C. Non-specific binding was determined in the presence of 10 μM DADLE. Separation of bound and free radioactivity was performed by filtration through Whatman GF/B filters in Brandel cell harvester, the filters were washed three times with 3 ml of ice-cold incubation buffer and radioactivity was determined by liquid scintillation. Binding data were fitted with the rectangular hyperbola and K_d_ and B_max_ values determined by GraphPad Prism4.

### Steady-state anisotropy of fluorescence of hydrophobic membrane probe DPH

Percoll-purified PM were labeled with diphenylhexatriene (DPH) by the fast addition (under mixing) of 1 mM stock solution of DPH in freshly distilled acetone to the membrane suspension (0.1 mg protein × ml^-1^; 1 μM final concentration); after 30 min at 25°C, which was allowed to ensure the optimum incorporation of the probe into the membrane interior [26], the anisotropy of DPH fluorescence was measured at Ex 365 nm/Em 425 nm wavelengths. Under these conditions, the fluorescence intensity of the membrane-bound DPH was ≈500x higher than that of the unbound free probe in aqueous medium; light scattering problems could be therefore omitted. Steady-state fluorescence anisotropy, r_DPH_, was calculated according to the formula: *r*_*DPH*_ = (*I*_*vv*_ − *I*_*vh*_)/(*I*_*vv*_ + 2*I*_*vh*_) as described before [27,28].

### The time-resolved fluorescence and dynamic depolarization of DPH

Fluorescence lifetime and polarization experiments were performed in a time correlated single photon counting (TCSPC) spectrofluorometer IBH 5000 U equipped with a cooled Hamamatsu R3809U-50 micro-channel plate photomultiplier detector as described before [29]. The sample was excited at 373 nm with a diode laser (IBH NanoLED-375L, FWHM 80 ps, 1 MHz repetition rate). The emission monochromator was set to 450 nm with slits set to 16 nm. A 400 nm cut-off filter was placed in the emission part of the instrument to reduce a parasitic light from the laser source. The sample was continuously mixed and kept at the constant temperature of 25°C. Measurement consisted in recording of fluorescence decays at 4 different orientations of the emission and excitation polarizers. The anisotropy free decay *I (t)*, which we used for the lifetime determination, was calculated according to Eq (1):

$$I(t)=I_{vv}(t)+2GI_{vh}(t)$$ (1)

where *I*_*vv*_ is the fluorescence decay measured with both excitation and emission polarized vertically, and *I*_*vh*_ with the vertically polarized excitation and horizontally polarized emission.

The G-factor (*G*) was determined by measuring a standard solution of POPOP and calculated according to Eq (2):

$$G=\frac{{\langle I_{hv}(t)\rangle }_{t}}{{\langle I_{hh}(t)\rangle }_{t}}\;,$$ (2)

where *I*_*hv*_ corresponds to the signal measured with the horizontally polarized excitation and vertically polarized emission, and *I*_*hh*_ to the excitation and emission both polarized horizontally. In order to obtain fluorescence lifetimes, the *I(t)* was fitted with a two-exponential decay (Eq 3):

$$I(t)=B_{1}\;\text{exp}(-t/\tau_{1})+B_{2}\;\text{exp}(-t/\tau_{2})\;,$$ (3)

yielding lifetimes *τ*_1_ and *τ*_2_ and corresponding amplitudes *B*_1_ and *B*_2_.

The decay of the anisotropy *r*(t) was determined according to Eq (4):

$$r(t)=\frac{I_{vv}(t)-GI_{vh}(t)}{I_{vv}(t)+2GI_{vh}(t)}\;,$$ (4)

and fitted with the formula (Eq 5):

r(t)=(r(0)−r(∞)).exp(−t/ϕ)+r(∞), (5)

where *r*(0), and *r*(∞) stands for the limiting and residual anisotropy, respectively and *ϕ* is the rotational correlation time. The anisotropy decays were fitted by the non-linear least squares method including the impulse re-convolution with the instrumental response function (fwhm ~ 100 ps). χ2 generated by the IBH software package served as goodness of fit criterion.

The anisotropy data were analyzed according to the “wobble in cone” model introduced by [30,31]. Wobbling diffusion constant (*D*_*w*_) and S-order parameter *S* were calculated according to Eqs 6 and 7,

$$D_{w}=\frac{\sigma_{s}}{\phi }\;,$$ (6)

S=(r(∞)r(0))12, (7)

where *r*(0), and *r*(∞) stand for limiting and residual anisotropy, *ϕ* is the rotational correlation time and *σ*_*s*_ is the relaxation time which is a function of the S-order parameter.

### Protein determination

Lowry method was used for determination of protein with serum albumin (fraction V, Sigma) as protein standard. The data were calculated by fitting the calibration curve as a quadratic equation.

## Results

### δ-OR-G protein coupling in low-density membranes prepared from PTX-untreated δ-OR-G_i_1α (C^351^I)-HEK293 cells at 0°C; effect of non-ionic detergent Brij-58

Cell homogenate was prepared from HEK293T cells stably expressing δ-OR-G_i_1α (C^351^I) fusion protein as described in Methods and divided by volume into 4 identical parts. The three parts were treated with increasing concentrations of Brij-58 0.025%, 0.05% and 0.1% w/v, respectively; the fourth part represented the control sample of homogenate containing no detergent. Subsequently, these 4 parts of homogenate, containing exactly the same protein concentration, were fractionated in parallel by flotation in sucrose density gradients for 24 hours and the basal and DADLE-stimulated, high-affinity [^32^P]GTPase was measured in gradient fractions (1–12) collected from the top to bottom of the centrifuge tube as described in Methods.

**Fig 1:**
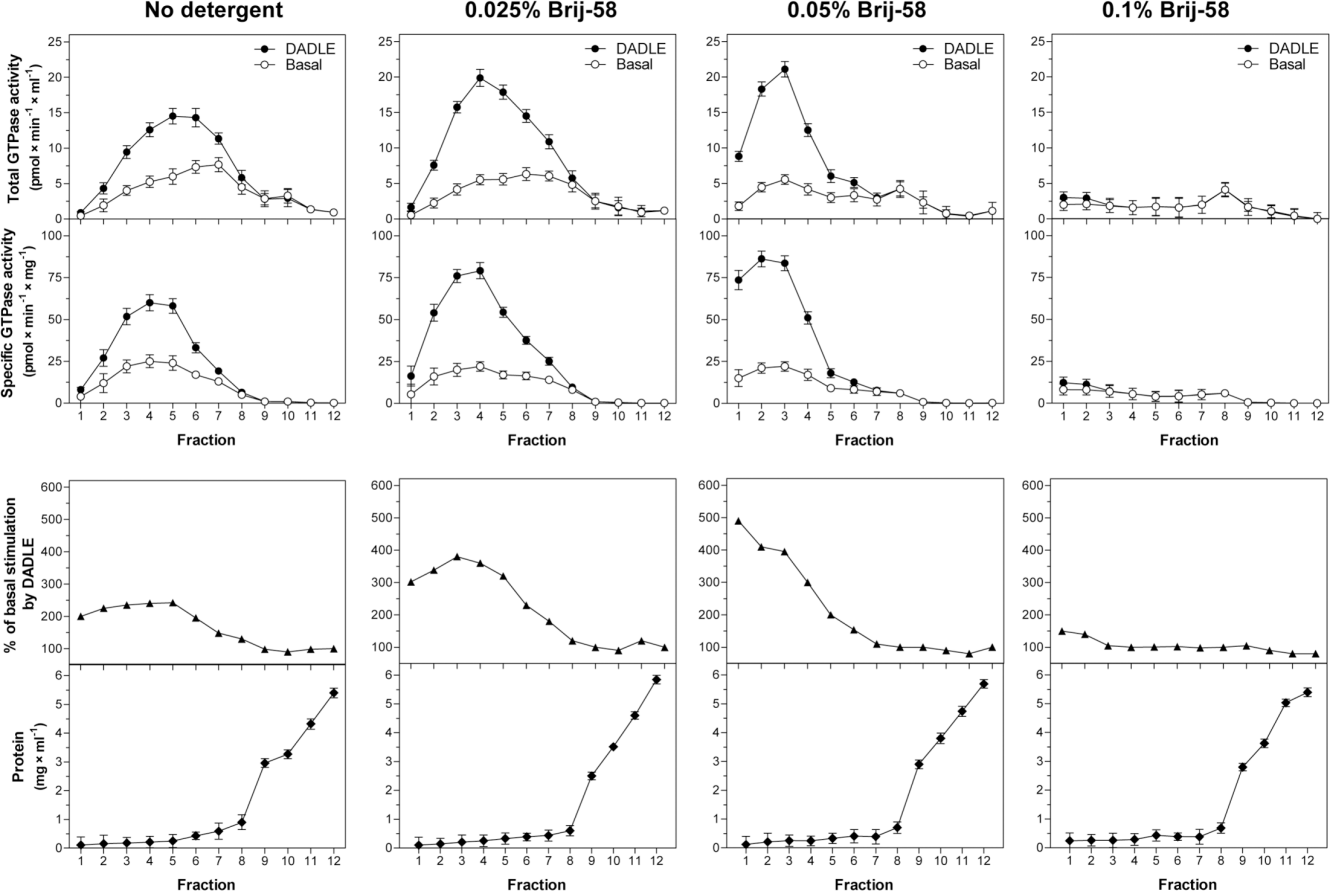
Subcellular fractionation of PTX-untreated δ-OR-G_i_1α (C^351^I)-HEK293 cells; density gradient profiles of basal and DADLE-stimulated [^32^P]GTPase activity. Cell homogenate prepared from δ-OR-G_i_1α cells was divided by volume into 4 identical parts. The three parts were treated with increasing concentrations of 0.025, 0.05 and 0.1% w/v Brij-58, respectively; the fourth part represented the control sample containing no detergent. Subsequently, these 4 parts of homogenate, containing exactly the same protein concentration, were fractionated by centrifugation in 15/20/25/30/35/40% w/v sucrose density gradient as described in Methods. **Upper panels.** Basal (○) and DADLE-stimulated (●), high-affinity [^32^P]GTPase activity was measured in gradient fractions 1–12 collected from top to the bottom of the centrifuge tube and expressed as total activity (pmol × min^-1^ × ml^-1^) or specific enzyme activity (pmol × min^-1^ × mg^-1^ protein) in each fraction. **Lower panels.** The ratio between the agonist-stimulated (DADLE) and basal level of specific enzyme activity was expressed as % of agonist stimulation over the basal level; the basal level represented 100%. The amount of protein in fractions was determined by Lowry method and expressed as mg per fraction (mg × ml^-1^). Results represent the average of 4 experiments ± SEM. The significance of difference between the specific DADLE-stimulated and basal [^32^P]GTPase activity in fractions 1–12 collected from sucrose density gradient 1 (no detergent), 2 (0.025% Brij-58), 3 (0.5% Brij-58) and 4 (0.1% Brij-58) was determined by Student´s t-test [see (A) in S1 Table]. Furthermore, net increment of agonist stimulation (Δ_DADLE_) was calculated as the difference between specific DADLE-stimulated and basal [^35^P]GTPase activity in fractions 1–6. The significance of difference of Δ_DADLE_ values in the four types of sucrose density gradients was determined by one-way ANOVA followed by Bonferroni´s multiple comparison test using GraphPad Prism4 [see (B) in S1 Table].

The high level of total [^32^P]GTPase activity per fraction (pmol × ml^-1^) and specific [^32^P]GTPase activity (pmol × min^-1^ × mg^-1^) was detected in “floating”, low-density fractions 1–6 (**Fig 1**). Membranes recovered in these fractions were collectively termed (in all 4 gradients) the low-density membranes, LDM. In LDM prepared in the absence of detergent (no detergent), DADLE-stimulation represented 180–240% of the basal level. In 0.025% Brij-58-treated LDM, agonist-stimulation was increased to 220–370%. The highest increase of DADLE stimulation (≈400%) was observed in 0.05% Brij-58-treated LDM. The further increase of detergent concentration to 0.1% was reflected in diminution of both basal and DADLE-stimulated [^32^P]GTPase activity to the zero level (**Fig 1**, right-hand panels). Net increment of DADLE-stimulated [^32^P]GTPase activity was also higher in 0.025% and 0.05% Brij-58-treated LDM when compared with LDM [see (B) in S1 Table].

**Fig 2:**
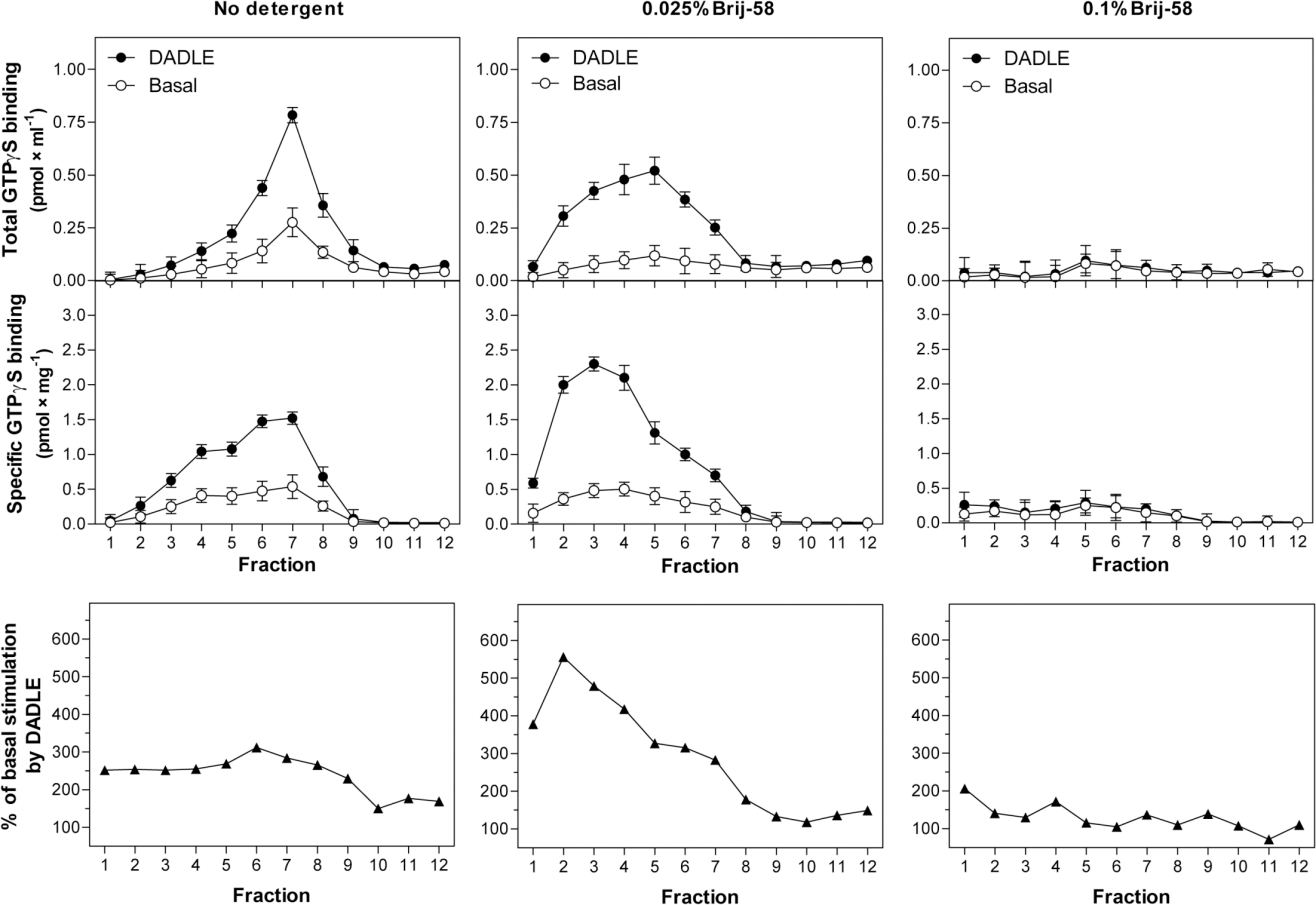
Subcellular fractionation of PTX-untreated δ-OR-G_i_1α (C^351^I)-HEK293 cells; density gradient profiles of basal and DADLE-stimulated [^35^S]GTPγS binding. Cell homogenate prepared from δ-OR-G_i_1α cells was divided by volume into 3 identical parts. The two parts were treated with 0.025% and 0.1% w/v Brij-58; the third part represented the control sample containing no detergent. Subsequently, these 3 parts of homogenate, containing exactly the same protein concentration, were fractionated by centrifugation in 15/20/25/30/35/40% w/v sucrose density gradients as described in Methods. **Upper panels.** Basal (○) and DADLE-stimulated (●) [^35^S]GTPγS binding was measured in fractions 1–12 and expressed as total binding (pmol × ml^-1^) or specific binding in each fraction (pmol × mg^-1^ protein). **Lower panels.** The ratio between the agonist-stimulated (DADLE) and the basal level of [^35^S]GTPγS binding was calculated from specific binding data (pmol × mg^-1^ protein) and expressed as % of agonist stimulation over the basal level; the basal level represented 100%. Results represent the average of 3 experiments ± SEM. The significance of difference between the specific DADLE-stimulated and basal [^35^S]GTPγS binding in fractions 1–12 collected from the three types of sucrose density gradients was determined by Student´s t-test [see (A) in S2 Table]. Net increment of agonist stimulation (Δ_DADLE_) was calculated as the difference between specific DADLE-stimulated and basal [^35^S]GTPγS binding in fractions 1–6. The significance of difference of Δ_DADLE_ values in the three types of sucrose density gradients was determined by one-way ANOVA followed by Bonferroni´s multiple comparison test using GraphPad Prism4 [see (B) in S2 Table].

Similar density gradient profiles were obtained by measurement of the high-affinity [^35^S]GTPγS binding in fractions 1–12 (**Fig 2**). The 0.025% Brij-58-treated LDM recovered in low-density fractions 1–6 exhibited ≈ 2-fold higher % of DADLE stimulation over the basal level when compared with the same fractions prepared from homogenate containing no detergent, LDM. The LDM isolated from homogenate treated with 0.1% Brij-58 exhibited the zero level of both basal and DADLE-stimulated [^35^S]GTPγS binding (**Fig 2**, right-hand panels). Net increment of DADLE-stimulated [^35^S]GTPγS binding was also significantly higher in 0.025% than in LDM [see (B) in S2 Table].

**Fig 3:**
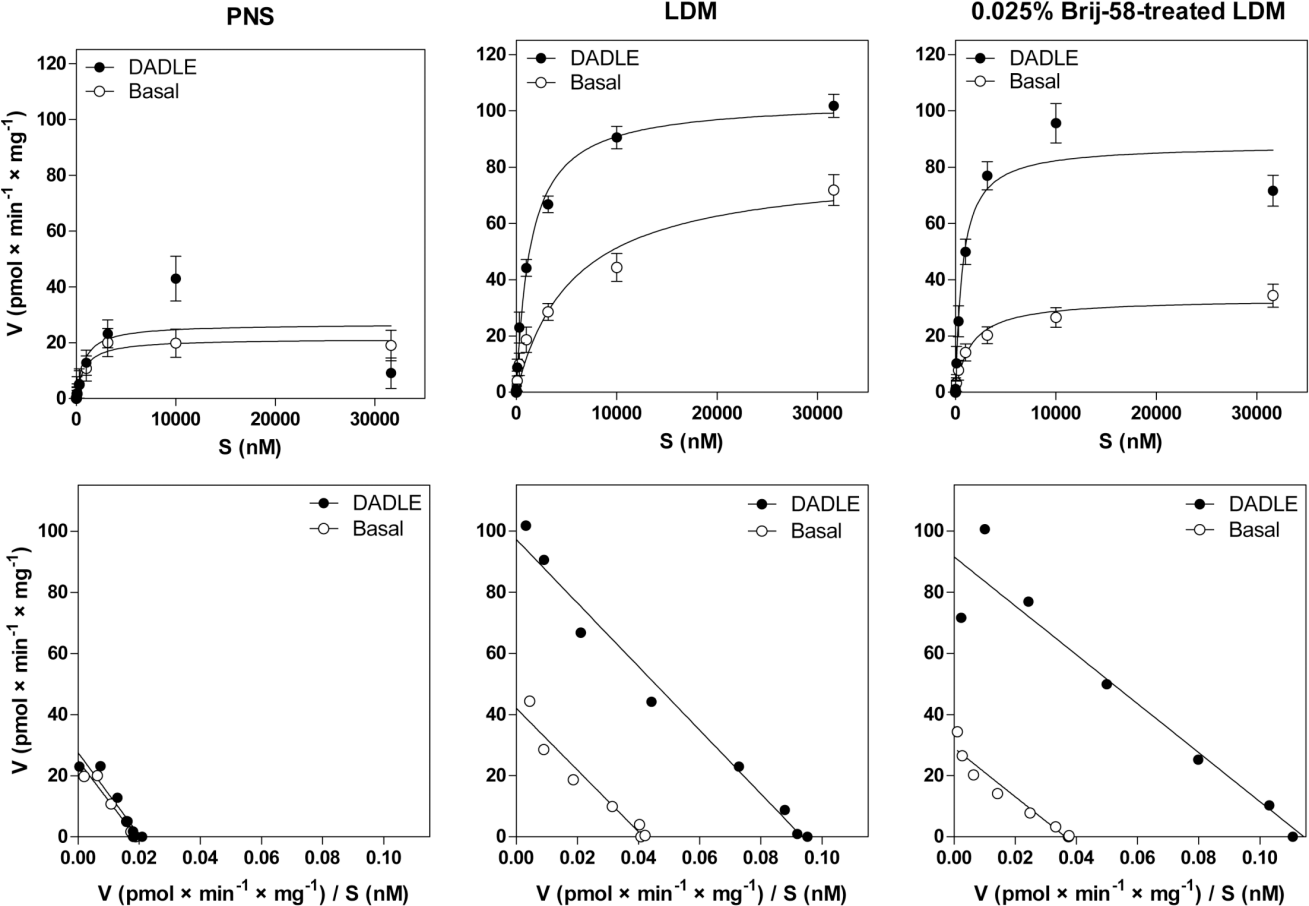
Basal and DADLE-stimulated [^32^P]GTPase activity in PNS, LDM and 0.025% Brij-58-treated LDM; determination of V_max_ and K_m_ values. Sucrose gradient fractions 1–6 prepared by fractionation of cell homogenate prepared from δ-OR-G_i_1α cells in the absence (LDM) or presence of 0.025% Brij-58 (0.025% Brij-58-treated LDM) were combined together, mixed and used for determination of basal and DADLE-stimulated [^32^P]GTPase as described in Methods. **Upper panels**. [^32^P]GTPase activity (pmol × min^-1^ × mg^-1^) was measured in the absence (○) or presence (●) of 100 μM DADLE at increasing concentrations of GTP. **Lower panels.** The data were expressed as Eadie-Hofstee plots and V_max_ (GTP) and Michaelis-Menten constant K_m_ (GTP) calculated by GraphPad Prism4. Results represent the average of 3 experiments ± SEM. The significance of difference of V_max_ and K_m_ values (between the basal and DADLE-stimulated [^32^P]GTPase) in PNS, LDM and 0.025% Brij-58-treated LDM was determined by Student´s t-test. The significance of difference of V_max_ and K_m_ values (for both basal and DADLE-stimulated [^32^P]GTPase) in PNS versus LDM versus 0.025% Brij-58-treated LDM was determined by one-way ANOVA followed by Bonferroni´s multiple comparison test using GraphPad Prism4 (see S3 Table).

Subsequently, the low-density fractions 1–6 were pooled together and the maximum enzyme activity V_max_ (GTP) and Michaelis-Menten constant K_m_ (GTP) of [^32^P]GTPase reaction were determined by substrate (GTP) saturation assay (**Fig 3**). As before, hydrolysis of [γ-^32^P]GTP was measured in the absence (basal) or presence of 100 μM DADLE. The maximum enzyme activity V_max_ (GTP) was increased by agonist from 42.1 ± 3.4 to 97.3 ± 3.7 pmol × min^-1^ × mg^-1^ in LDM and from 29.1 ± 2.0 to 91.5 ± 5.0 pmol × min^-1^ × mg^-1^ in 0.025% Brij-58-treated LDM (**Fig 3**, **Table 1**).

**Table 1:** DADLE-stimulated [^32^P]GTPase activity in membranes prepared from PTX-untreated cells; GTP saturation ± DADLE.

		PNS	LDM	0.025% Brij-58-treated LDM
**V**_**max**_	(-DADLE)	25.0	42.1*	29.1ND
	(+DADLE)	27.5	97.3***	91.5***
**Δ**_**max**_		2.5	55.2**	62.4**
**B**_**max,**_ **[** ^**3**^ **H]naltrindole**		0.56	3.61*	1.74**
**Δ**_**max**_ **/B**_**max**_		4.5	15.3*	35.9*
**K**_**m**_ **(GTP)**	(-DADLE)	1.3	1.0ND	0.8*
	(+DADLE)	1.4	1.0ND	0.8*

[^32^P]GTPase was measured at increasing concentrations of GTP and maximum enzyme activity V_max_ (pmol × min^-1^ × mg^-1^) and Michaelis-Menten constant K_m_ (nM) were calculated from Eadie-Hofstee plots presented in lower panels of **Fig 3**. Net increment of DADLE-stimulation **(**Δ_max_ **)** was determined as the difference between V_max_ (+DADLE) and V_max_ (-DADLE). B_max_, number of [^3^H]naltrindole binding sites (pmol × mg^-1^); Δ_max_/B_max_ ratio, Δ_max_ normalized to receptor number.

* (p<0.05)

** (p<0.01)

*** (p<0.001) indicates a significant difference between PNS and LDM (no detergent) or between PNS and 0.025% Brij-58-treated LDM; ND (p>0.05), not different.

The net increment of agonist stimulation Δ_max,_ calculated as the difference between V_max_ of DADLE-stimulated (+DADLE) and V_max_ of basal level (-DADLE) of [^32^P]GTPase activity was in LDM 22-fold higher than in post-nuclear fraction (PNS); in Brij-58-treated LDM, Δ_max_ was 25-fold higher than in PNS (**Table 1**). Thus, the significant purification of δ-OR-G_i_1α (C^351^I) fusion protein in both preparations of LDM was obtained. Determination of K_m_ values in PNS ± DADLE (1.3 ± 0.1 and 1.4 ± 0.2 μM), in LDM ± DADLE (1.0 ± 0.1 and 1.0 ± 0.1 μM) and in 0.025% Brij-58-treated LDM ± DADLE (0.8 ± 0.1 and 0.8 ± 0.1 μM) indicated the same affinity of [^32^P]GTPase to its substrate and no change by an agonist.

**Fig 4:**
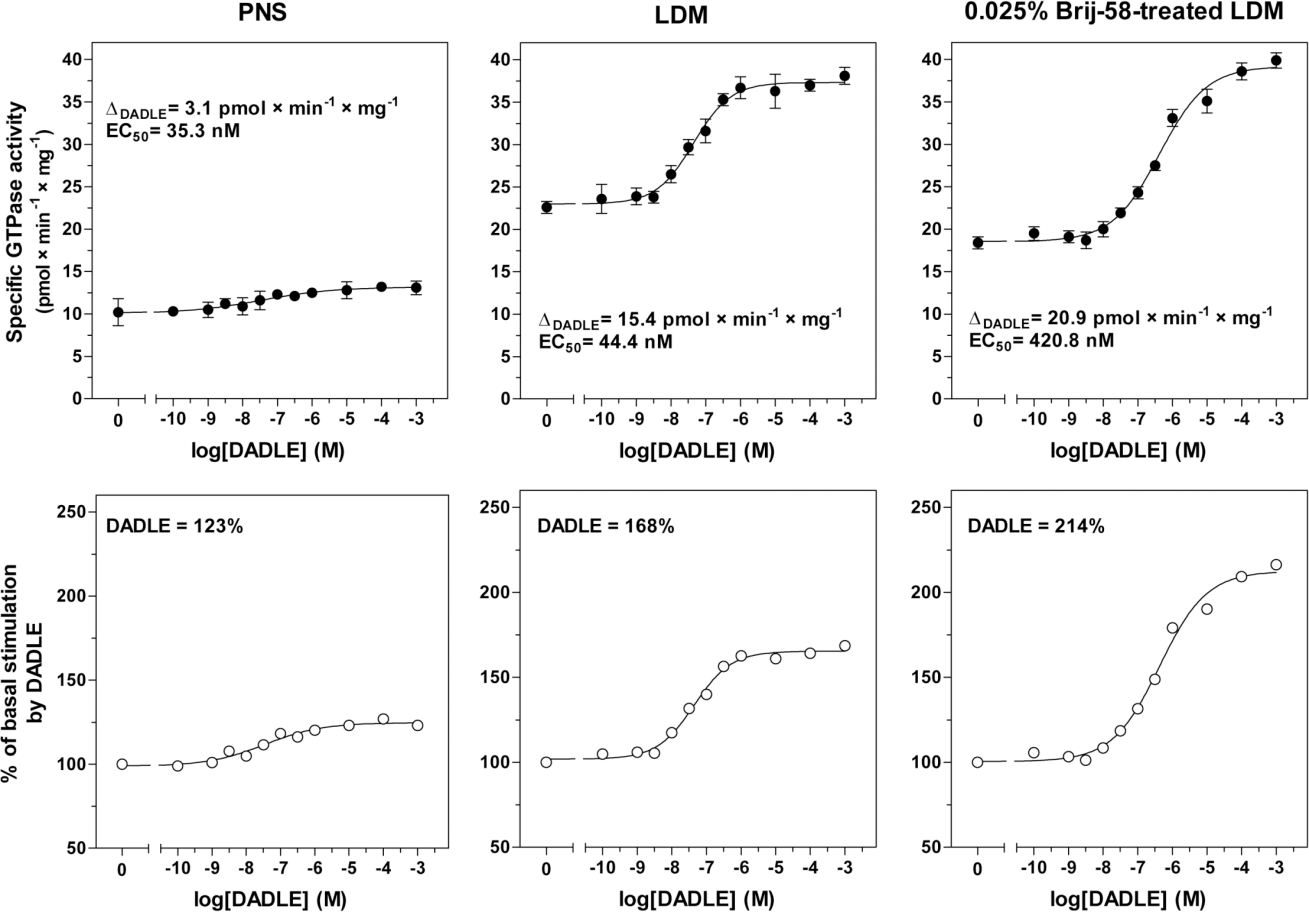
Dose-response curves of DADLE-stimulated [^32^P]GTPase; comparison of PNS, LDM and 0.025% Brij-58-treated LDM. **Upper panels**. Dose-response curves of DADLE-stimulated [^32^P]GTPase were measured in post-nuclear supernatant (PNS), low-density membranes (LDM) and 0.025% Brij-58-treated LDM prepared from δ-OR-G_i_1α cells as described in Methods. LDM and 0.025% Brij-58-treated LDM represent the mixed pool of membranes in combined fractions 1–6 prepared in the absence or presence of detergent, respectively. The net increment of agonist-stimulation (Δ_DADLE_) and DADLE concentration inducing the half-maximum response (EC_50_) were calculated by GraphPad Prism4. **Lower panels.** The ratio between the agonist-stimulated (DADLE) and basal level of specific enzyme activity was expressed as % of agonist stimulation over the basal level; the basal level represented 100%. Results represent the average of 3 experiments ± SEM. The significance of difference of EC_50_ and Δ_DADLE_ in PNS versus LDM versus 0.025% Brij-58-treated LDM was determined by one-way ANOVA followed by Bonferroni´s multiple comparison test using GraphPad Prism4 (see S4 Table).

The results of measurement of maximum enzyme activity ± DADLE described in the previous paragraph were supported by comparison of the dose-response curves of DADLE-stimulated [^32^P]GTPase in PNS, LDM and 0.025% Brij-58-treated LDM (**Fig 4**). DADLE-stimulated [^32^P]GTPase measured at saturating concentration of agonist (v_DADLE_) was substantially increased by purification of LDM: the net increment of agonist stimulation (Δ_DADLE_) was increased from 3.1 ± 0.3 pmol × min^-1^ × mg^-1^ in PNS to 15.4 ± 0.5 pmol × min^-1^ × mg^-1^ in LDM. In 0.025% Brij-58-treated LDM, Δ_DADLE_ (20.9 ± 0.7 pmol × min^-1^ × mg^-1^) was 7-fold higher than in PNS (**Fig 4**, upper panels). However, the potency of [^32^P]GTPase activation by DADLE, expressed as the agonist concentration inducing the half-maximum response (EC_50_), was decreased by one order of magnitude in 0.025% Brij-58-treated LDM (EC_50_ = 420.8 ± 2.0 nM) when compared with LDM (EC_50_ = 44.4 ± 2.1 nM) or PNS (35.3 ± 2.0 nM) (**Fig 4** and **Table 2**).

**Table 2:** DADLE-stimulated [^32^P]GTPase activity in membranes prepared from PTX-untreated cells; dose-response curves.

	PNS	LDM	0.025% Brij-58-treated LDM
**Δ**_**DADLE**_	3.1	15.4*	20.9**
**Δ**_**DADLE**_ **/B**_**max**_	5.5	4.3ND	12.0*
**EC**_**50**_	35.3	44.4ND	420.8***

[^32^P]GTPase activity was measured in the absence (V_basal_) or presence (V_DADLE_) of increasing concentrations of DADLE (**Fig 4**). Δ_DADLE_, net increment of agonist stimulation, was calculated as the difference between V_DADLE_ at saturating concentration and V_basal_; Δ_DADLE_/B_max_ ratio, Δ_DADLE_ normalized to receptor number; EC_50_ (nM); DADLE concentration inducing half-maximum stimulation of [^32^P]GTPase activity.

* (p<0.05)

** (p<0.01)

*** (p<0.001) indicates a significant difference between PNS and LDM (no detergent) or between PNS and 0.025% Brij-58-treated LDM; ND (p>0.05), not different.

**Fig 5:**
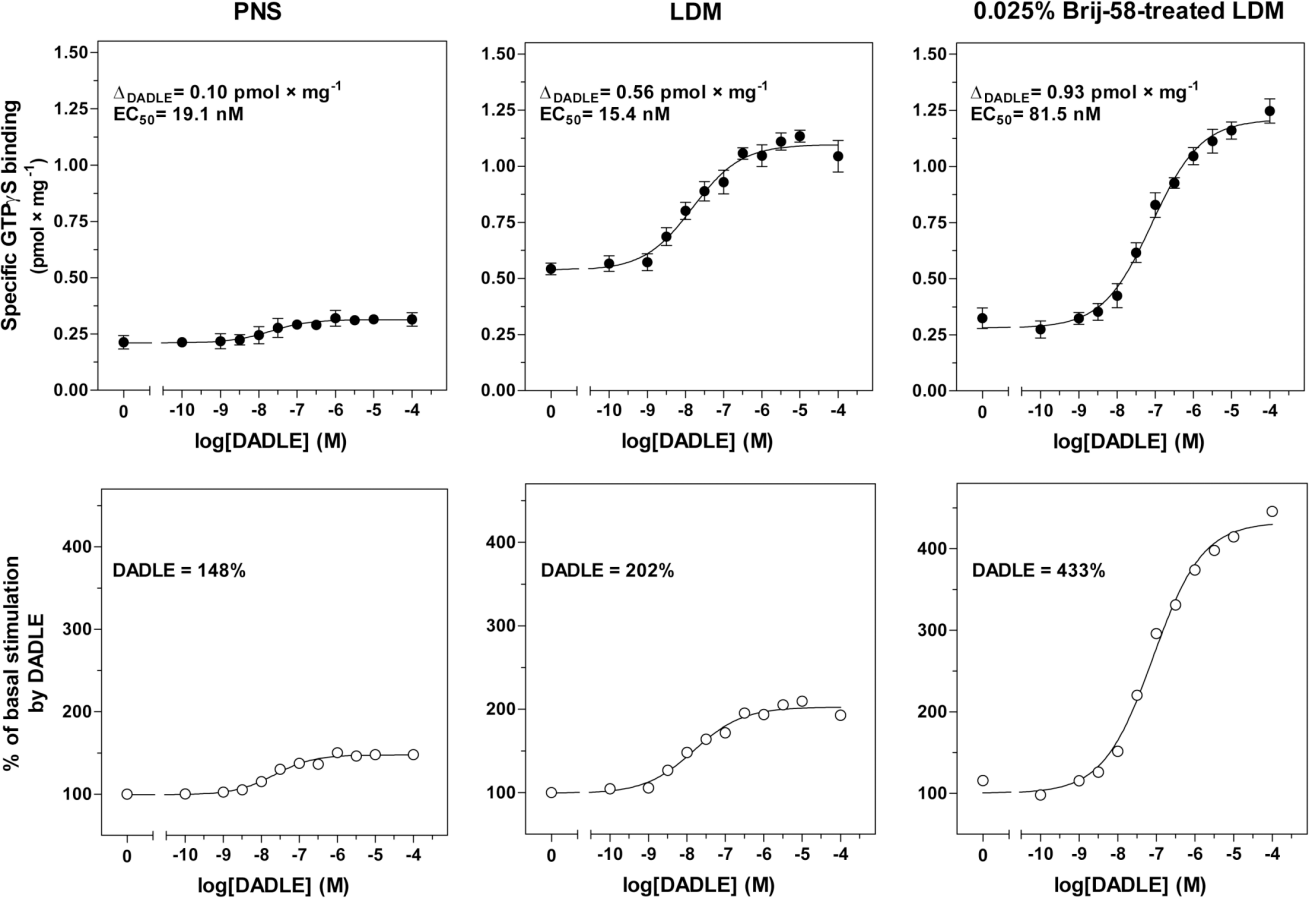
Dose-response curves of DADLE-stimulated [^35^S]GTPγS binding; comparison of PNS, LDM and 0.025% Brij-58-treated LDM. **Upper panels**. Dose-response curves of DADLE-stimulated [^35^S]GTPγS binding were measured in PNS, LDM and 0.025% Brij-58-treated LDM as described in Methods. LDM and 0.025% Brij-58-treated LDM represent the mixed pool of membranes in combined fractions 1–6 prepared from δ-OR-G_i_1α cells in the absence or presence of detergent, respectively. The net increment of agonist-stimulation (Δ_DADLE_) and DADLE concentration inducing the half-maximum response [EC_50_ (DADLE)] were determined by GraphPad Prism4. **Lower panels.** The ratio between the agonist-stimulated (DADLE) and the basal level of [^35^S]GTPγS binding was expressed as % of agonist stimulation over the basal level; the basal level represented 100%. Results represent the average of 3 experiments ± SEM. The significance of difference of EC_50_ and Δ_DADLE_ in PNS versus LDM versus 0.025% Brij-58-treated LDM was determined by one-way ANOVA followed by Bonferroni´s multiple comparison test using GraphPad Prism4 (see S5 Table).

The significant increase of purity of δ-OR-G_i_1α in LDM and Brij-58-treated LDM, which was, in the case of Brij-58-treated LDM, accompanied by a decrease of potency of G protein response (5-fold), was also detected by comparison of the dose-response curves of DADLE-stimulated [^35^S]GTPγS binding (**Fig 5**, **Table 3**). The net increment of agonist stimulation in LDM (Δ_DADLE_ = 0.56 ± 0.07 pmol × mg^-1^) and 0.025% Brij-58-treated LDM (Δ_DADLE_ = 0.93 ± 0.08 pmol × mg^-1^) was 6- and 9-fold higher than in PNS (0.10 ± 0.05 pmol × mg^-1^). As before, EC_50_ values were not significantly different when compared in PNS (19.1 ± 1.3 nM) and LDM (15.4 ± 1.4 nM), but significantly increased in Brij-58-treated LDM (EC_50_ = 81.5 ± 3.4 nM). Thus, the lower potency of agonist response in Brij-58-treated LDM was noticed by measurement of both DADLE-stimulated, high-affinity [^32^P]GTPase (**Fig 4**) and [^35^S]GTPγS binding (**Fig 5**).

**Table 3:** DADLE- stimulated [^35^S]GTPγS binding in membranes prepared from PTX-untreated cells; dose-response curves.

	PNS	LDM	0.025% Brij-58-treated LDM
**Δ**_**DADLE**_	0.10	0.56**	0.93***
**Δ**_**DADLE**_ **/B**_**max**_	0.18	0.16ND	0.53**
**EC**_**50**_	19.1	15.4ND	81.5**

Δ_DADLE_ (pmol × mg^-1^), net increment of agonist stimulation at saturating concentration of DADLE; Δ_DADLE_/B_max_ ratio, Δ_DADLE_ normalized to receptor number; EC_50_ (nM), DADLE concentration inducing half-maximum stimulation of [^35^S]GTPγS binding.

* (p<0.05)

** (p<0.01)

*** (p<0.001) indicates a significant difference between PNS and LDM or 0.025% Brij-58-treated LDM; ND (p>0.05), not different.

**Fig 6:**
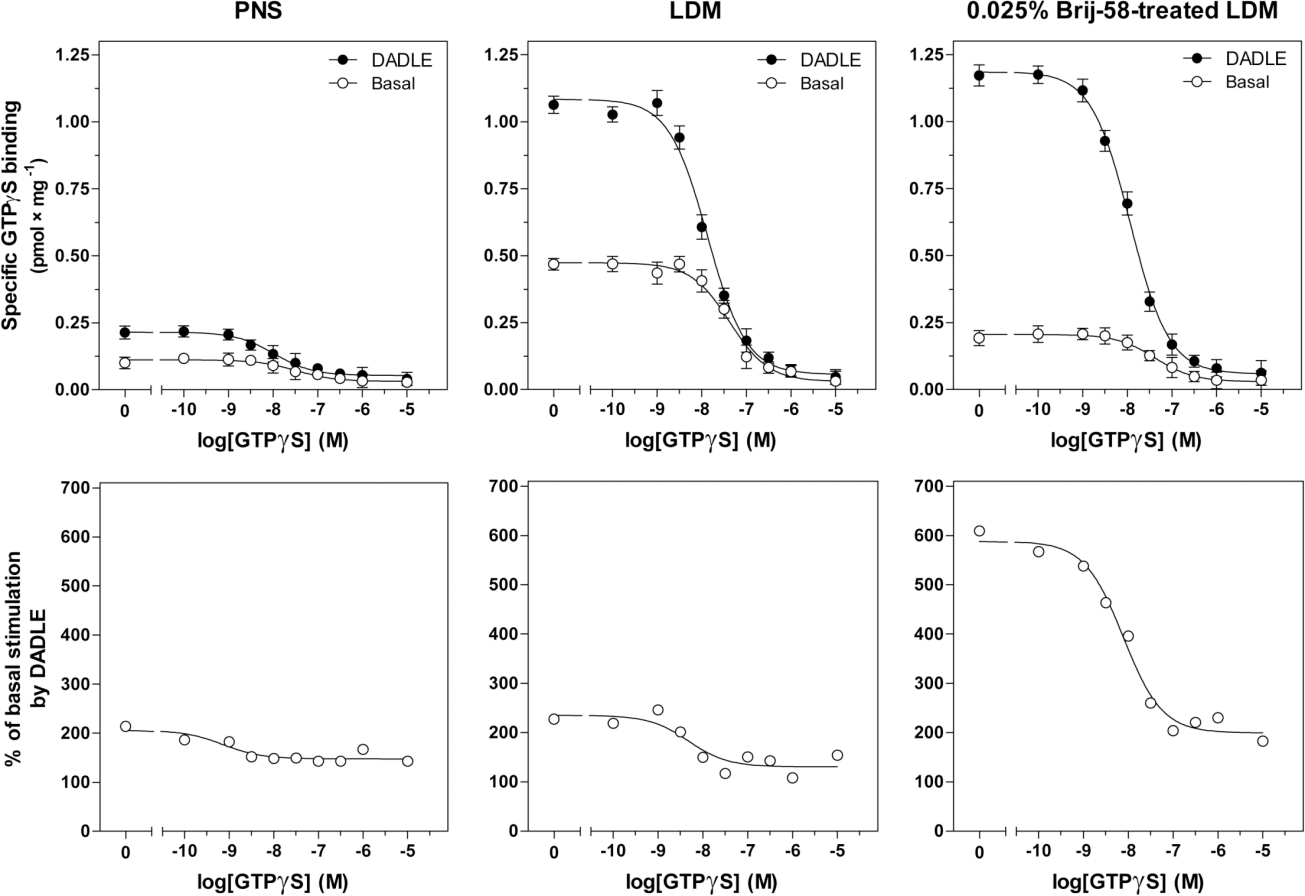
Competitive [^35^S]GTPγS/GTPγS binding curves; comparison of PNS, LDM and 0.025% Brij-58-treated LDM. **Upper panels**. [^35^S]GTPγS binding to PNS, LDM and 0.025% Brij-58-treated LDM prepared from δ-OR-G_i_1α cells was measured in absence (B_0_) or presence of increasing concentrations of unlabeled GTPγS as described in Methods; IC_50_ values were calculated by GraphPad Prism4. **Lower panels.** The ratio between the agonist-stimulated (DADLE) and the basal level of [^35^S]GTPγS binding was expressed as % of agonist stimulation over the basal level; the basal level represented 100%. The data represent the average of 3 experiments ± SEM. The significance of difference of IC_50_ and ΔB_0_ values in PNS versus LDM versus 0.025% Brij-58-treated LDM was determined one-way ANOVA followed by Bonferroni´s multiple comparison test using GraphPad Prism4 (see S6 Table).

In the following step of our work, PNS, LDM and 0.025% Brij-58-treated LDM were compared by measurement of [^35^S]GTPγS/GTPγS competitive inhibition curves determined in the presence or absence of 100 μM DADLE (**Fig 6**). Purification of LDM by flotation in sucrose gradient and increase of efficacy by detergent was documented again. The DADLE-stimulated [^35^S]GTPγS binding level at zero concentration of GTPγS (B_0_), was increased from 0.22 ± 0.01 pmol × mg^-1^ in PNS to 1.08 ± 0.02 and 1.19 ± 0.02 pmol × mg^-1^ in LDM and 0.025% Brij-58-treated LDM, respectively. The net increment of agonist stimulation (ΔB_0_), calculated as the difference between B_0_ (+DADLE) and B_0_ (—DADLE), was increased from 0.11 ± 0.01 pmol × mg^-1^ in PNS to 0.61 ± 0.03 pmol × mg^-1^ and 0.98 ± 0.02 pmol × mg^-1^ in LDM and Brij-58-treated LDM, respectively (**Table 4**). Comparison of IC_50_ values in PNS, LDM and Brij-58-treated LDM indicated no significant difference between the three types of membranes in this type of experiment (**Fig 6**).

**Table 4:** DADLE-stimulated [^35^S]GTPγS binding in membranes prepared from PTX-untreated cells; [^35^S]GTPγS/GTPγS competition binding curves.

		PNS	LDM	0.025% Brij-58-treated LDM
**(-DADLE)**				
	**B**_**0**_	0.11	0.47**	0.21*
	**B**_**0**_ **(-DADLE)/B**_**max**_	0.20	0.13ND	0.12ND
	**IC**_**50**_	32.7	42.3ND	41.9ND
**(+DADLE)**				
	**B**_**0**_	0.22	1.08**	1.19**
	**B**_**0**_ **(+DADLE)/B**_**max**_	0.39	0.30ND	0.68*
	**IC**_**50**_	10.6	13.5ND	11.6ND
**ΔB**_**0**_		0.11	0.61**	0.98***
**ΔB**_**0**_ **/B**_**max**_		0.20	0.17ND	0.56*

B_0_ (pmol × mg^-1^), [^35^S]GTPγS binding in the absence of GTPγS; ΔB_0_, the difference between B_0_ (+DADLE) and B_0_ (-DADLE); ΔB_0_/B_max_ ratio, ΔB_0_ normalized to receptor number; IC_50_ (nM), GTPγS concentration inducing half-maximum inhibition of [^35^S]GTPγS binding.

* (p<0.05)

** (p<0.01)

*** (p<0.001) indicates a significant difference between PNS and LDM or 0.025% Brij-58-treated LDM; ND (p>0.05), not different.

**Fig 7:**
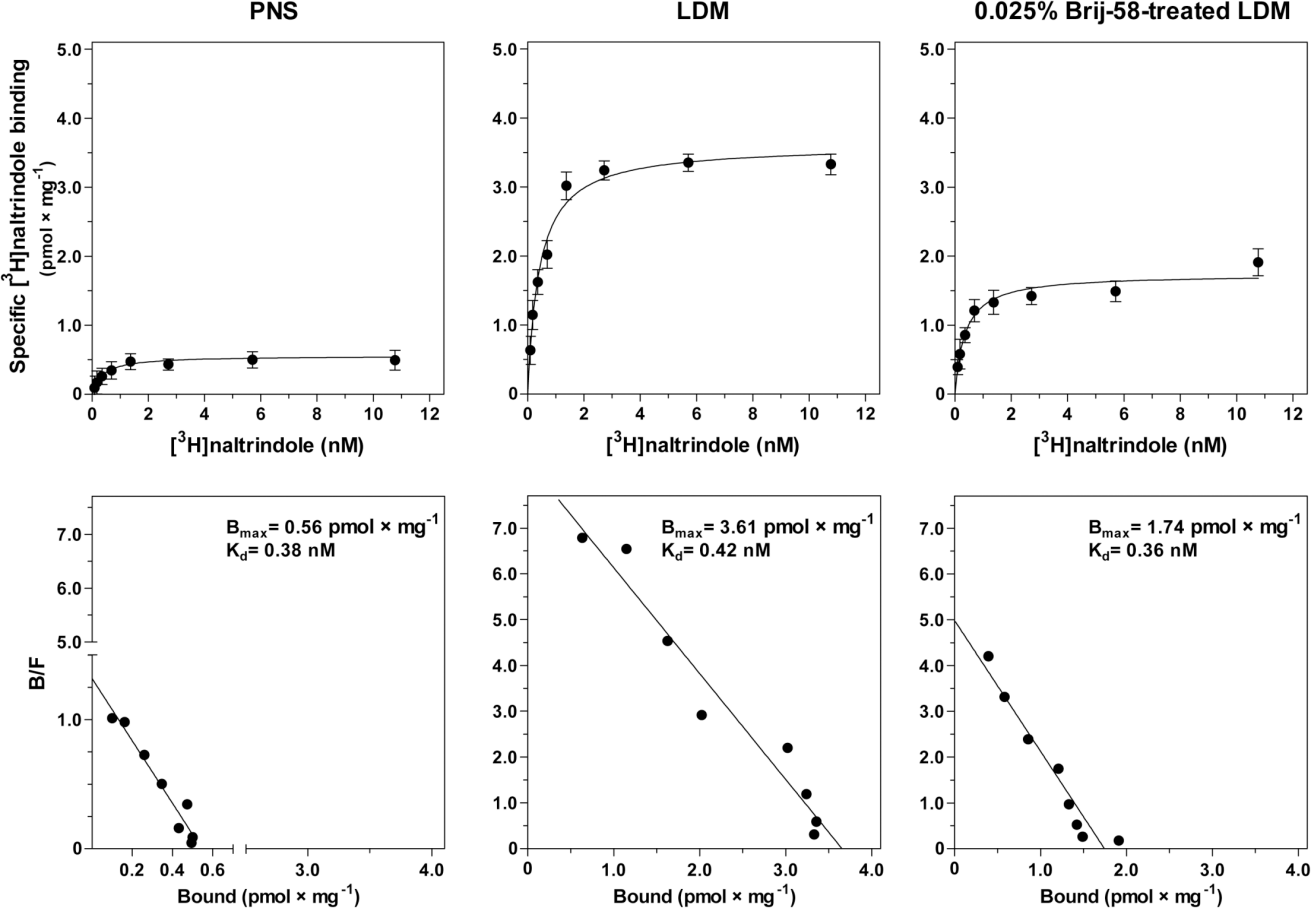
Saturation of [^3^H]naltrindole binding sites in PNS, LDM and 0.025% Brij-58-treated LDM. Saturation binding studies of specific δ-opioid antagonist [^3^H]naltrindole sites in PNS, LDM and 0.025% Brij-58-treated LDM was measured as described in Methods. The B_max_ and K_d_ values were calculated by GraphPadPrism4. The data represent the average of 3 experiments ± SEM. Significance of difference of B_max_ and K_d_ values in PNS versus LDM versus 0.025% Brij-58-treated LDM was determined one-way ANOVA followed by Bonferroni´s multiple comparison test using GraphPad Prism4 (see S7 Table).

We have also determined the number of δ-opioid receptors in PNS, LDM and 0.025% Brij-58-treated LDM by saturation binding assay with antagonist [^3^H]naltrindole (**Fig 7**). As before, purification of δ-OR-G_i_1α protein was evidenced again because B_max_ of [^3^H]naltrindole binding in LDM (3.61 ± 0.21 pmol × mg^-1^) was 7-fold higher than in PNS (0.56 ± 0.21 pmol × mg^-1^). However, in 0.025% Brij-58-treated LDM, number of receptor sites was less than half of this amount (B_max_ = 1.74 ± 0.11 pmol × mg^-1^). This result may be interpreted as the detergent-induced loss of approximately half of membrane bound δ-OR. The affinity of [^3^H]naltrindole binding was unchanged by detergent treatment.

The B_max_ values of [^3^H]naltrindole binding were subsequently used for normalization of [^32^P]GTPase results according to the number of receptors present in a given membrane preparation. Calculation of Δ_max_/B_max_ and Δ_DADLE_/B_max_ ratios in Brij-58-treated LDM indicated 2.4- and 2.8-fold higher values than in LDM (**Tables 1 and 2**). Similar ratios were obtained by normalization of [^35^S]GTPγS binding data: Δ_DADLE_/B_max_ and ΔB_0_/B_max_ ratios were 3.3-fold higher in Brij-58-treated LDM than in LDM (**Tables 3 and 4**). These results may be simply interpreted as a loss of about half of receptor sites in 0.025% Brij-58-treated LDM when compared with LDM.

It may be therefore concluded that Brij-58-treated LDM prepared at a low detergent concentrations exhibit the high efficacy when activating their cognate G proteins, but the affinity of agonist response, expressed as EC_50_ values of DADLE-stimulated [^32^P]GTPase or [^35^S]GTPγS binding, is decreased by one order of magnitude.

### δ-OR-G protein coupling in low-density membranes prepared from PTX-treated δ-OR-G_i_1α (C^351^I)-HEK293 cells at 0°C; effect of non-ionic detergent Brij-58

**Fig 8:**
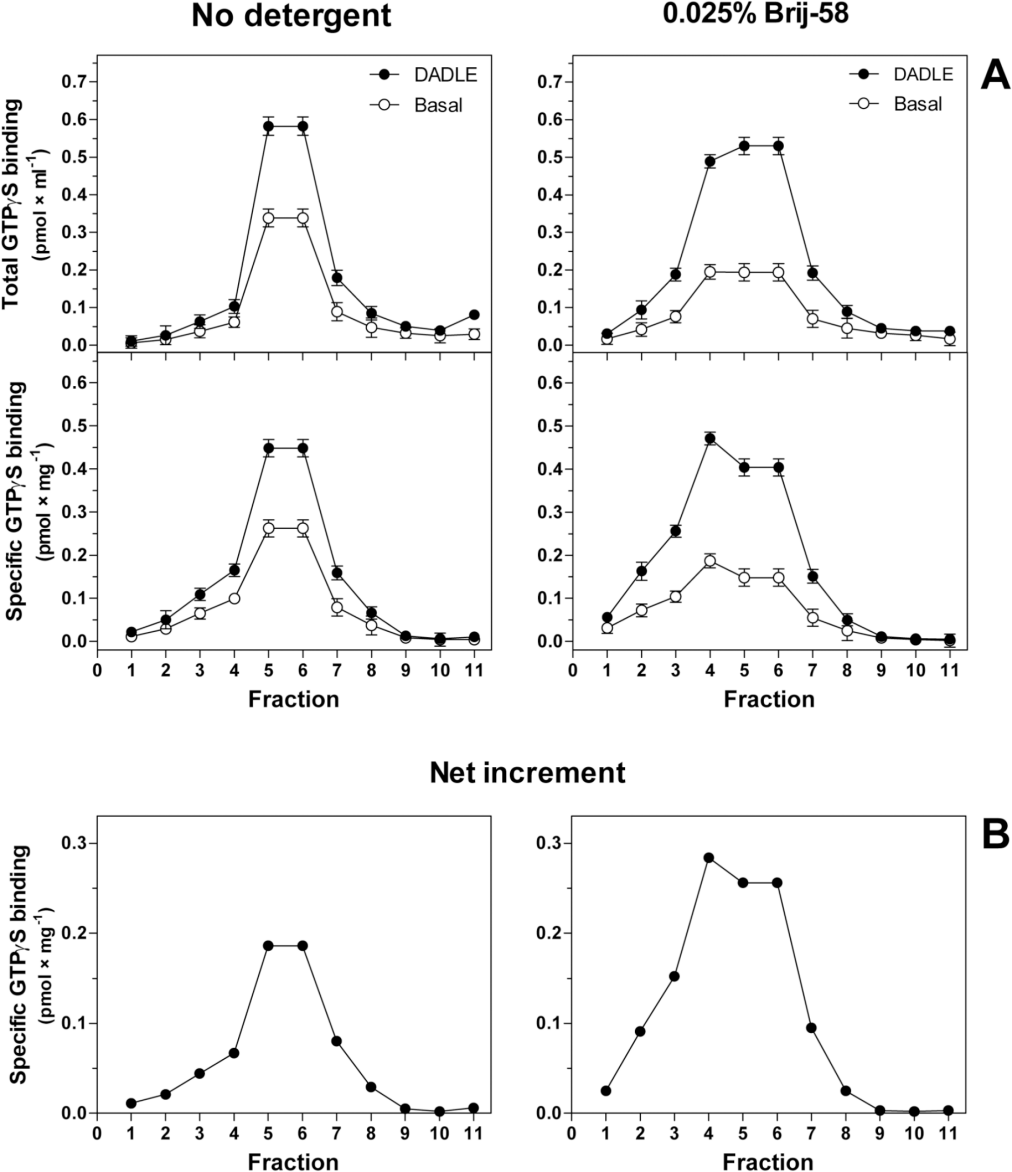
Subcellular fractionation of PTX-treated δ-OR-G_i_1α cells; density gradient profiles of basal and DADLE-stimulated [^35^S]GTPγS binding. Pertussis toxin-treated δ-OR-G_i_1α cells were homogenized and fractionated in the absence (no detergent) or presence of 0.025% Brij-58 in sucrose gradient as in studies of PTX-untreated cells (see Methods). (**A**) Basal (○) and DADLE-stimulated (●) [^35^S]GTPγS binding was measured in fractions collected from the top to bottom of the centrifuge tube and expressed as total (pmol × ml^-1^) or specific binding (pmol × mg^-1^) in a given fraction. (**B**) Net increment of DADLE stimulation (Δ_DADLE_) was calculated as the difference between specific DADLE-stimulated and basal level of [^35^S]GTPγS binding and expressed as pmol × mg^-1^. Results represent the average of 3 experiments ± SEM. The significance of difference between the specific DADLE-stimulated and basal [^35^S]GTPγS binding in fractions was determined by Student´s t-test (see S8 Table).

Virtually the same results were obtained in analysis of 0.025% Brij-58-treated membranes prepared from δ-OR-G_i_1α cells exposed to pertussis toxin (10 ng × ml^-1^) for 12 hours. The screening of density gradient profiles indicated that the peak of DADLE-stimulated [^35^S]GTPγS binding was shifted to lower densities: from fractions 5+6, which were combined together and corresponded to plasma membrane fractions, to fraction 4. The net increment of agonist stimulation (Δ_DADLE)_ in fractions 5+6 was increased by Brij-58 from 0.19 ± 0.02 to 0.26 ± 0.02 pmol × mg^-1^. In fraction 4, Δ_DADLE_ was increased from 0.07 ± 0.01 to 0.28± 0.02 pmol × mg^-1^ (**Fig 8**). The average net increment of agonist stimulation in combined fractions 4+5+6 was 1.8-fold higher than in the same fractions prepared in the absence of detergent.

**Fig 9:**
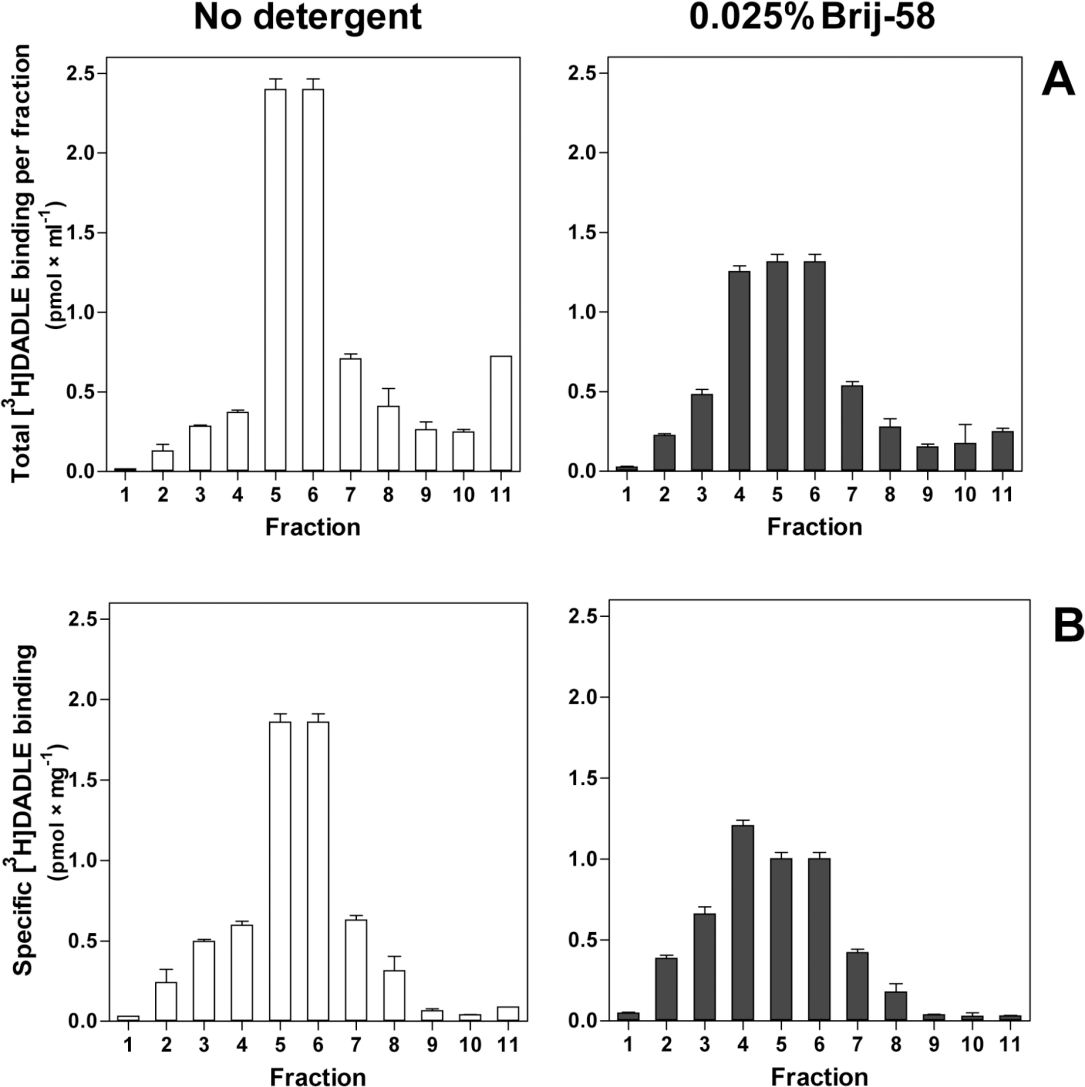
Density gradient profiles of [^3^H]DADLE binding; PTX-treated δ-OR-G_i_1α cells. PTX-treated δ-OR-G_i_1α cells were homogenized and fractionated as described in legend to Fig 8. **(A)** [^3^H]DADLE binding was determined in fractions as described in Methods and expressed as total binding (pmol × ml^-1^) or **(B)** specific binding (pmol × mg^-1^) in a given fraction at the saturating 10 nM concentration. Results represent the average of 3 experiments ± SEM. The significance of difference of [^3^H]DADLE binding between fractions collected from gradients containing no detergent and 0.025% Brij-58 was determined by Student´s t-test (see S9 Table).

In this set of experiments, we have also determined the δ-OR number by agonist binding assay using [^3^H]DADLE. As expected from the previous results of antagonist binding (**Fig 7**), the level of specific [^3^H]DADLE binding in 0.025% Brij-58-treated fractions 5–6 was ≈2-fold lower than in the same fractions prepared in the absence of detergent (**Fig 9**). The detergent-induced shift of receptors to lower densities was noticed as an increase of [^3^H]DADLE binding in fraction 4 from 0.60 ± 0.02 to 1.21 ± 0.03 pmol × mg^-1^ proceeding in parallel with the decrease of agonist binding in fractions 5–6 from 1.86 ± 0.03 to 1.00 ± 0.03 pmol × mg^-1^. The sum of [^3^H]DADLE binding in fractions 4, 5 and 6 was decreased by Brij-58 from 4.32 ± 0.04 pmol × mg^-1^ to 3.21 ± 0.04 pmol × mg^-1^. Normalization of the net increment of DADLE stimulation (Δ_DADLE_) to δ-OR number (B) in fractions 4–6, indicated the 2.4-fold higher Δ_DADLE_/B ratio in Brij-58-treated fractions than in the same fractions prepared in the absence of detergent (**Table 5)**.

**Table 5:** Net increment of DADLE-stimulated [^35^S]GTPγS binding and [^3^H]DADLE binding in sucrose fractions collected from PTX-treated δ-OR-G_i_1α cells.

	No detergent		0.025% Brij-58	
Fraction	4	5+6	4	5+6
**Δ**_**DADLE**_	0.07	0.19	0.28**	0.26*
**B**	0.60	1.86	1.21*	1.00*
**Δ**_**DADLE**_ **/B**	0.11	0.10	0.23	0.26

Δ_DADLE_ (pmol × mg^-1^), net increment of agonist stimulated [^35^S]GTPγS binding at 10 μM DADLE; B (pmol × mg^-1^), [^3^H]DADLE binding at saturating 10 nM concentration; Δ_DADLE_/B, ratio between Δ_DADLE_ and B.

* (p<0.05)

** (p<0.01) indicates a significant difference between the two types of membranes; ND (p>0.05), not different.

**Fig 10:**
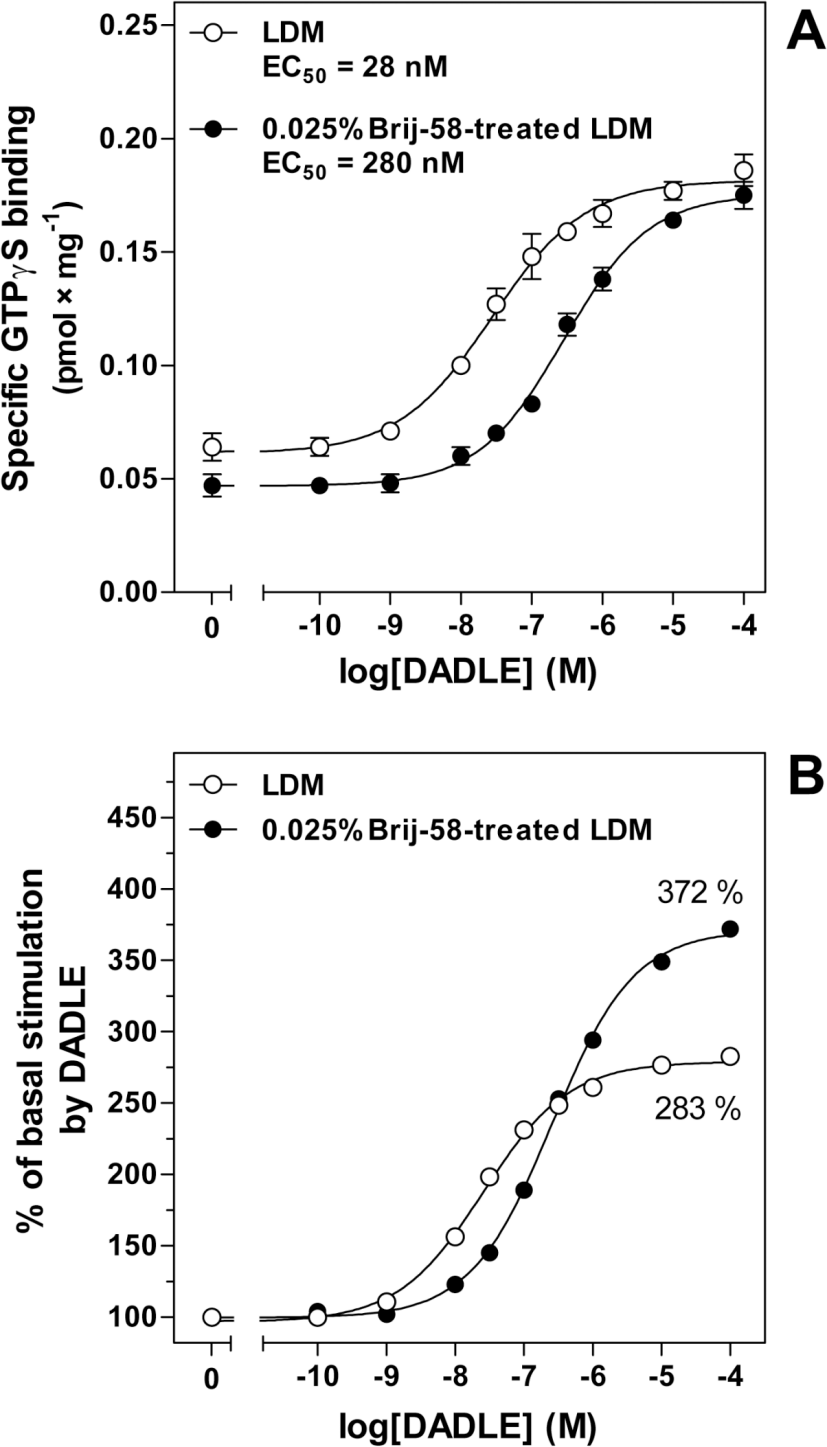
Dose-response curves of DADLE-stimulated [^35^S]GTPγS binding in LDM and 0.025% Brij-58-treated LDM prepared from PTX-treated δ-OR-G_i_1α cells. Sucrose gradient fractions 1–6 were combined together, mixed and used for determination of the dose-response curves of DADLE-stimulated [^35^S]GTPγS binding as described in Methods. **(A)**. DADLE concentration inducing the half-maximum response [EC_50_ (DADLE)] was determined by GraphPad Prism4. **(B)** The ratio between the DADLE-stimulated and basal level of [^35^S]GTPγS binding was expressed as % of agonist stimulation over the basal level; the basal level represented 100%. Results represent the average of 3 experiments ± SEM. LDM, fractions collected from gradient containing no detergent; 0.025% Brij-58-treated LDM, fractions collected from gradient containing 0.025% Brij-58.

**Fig 11:**
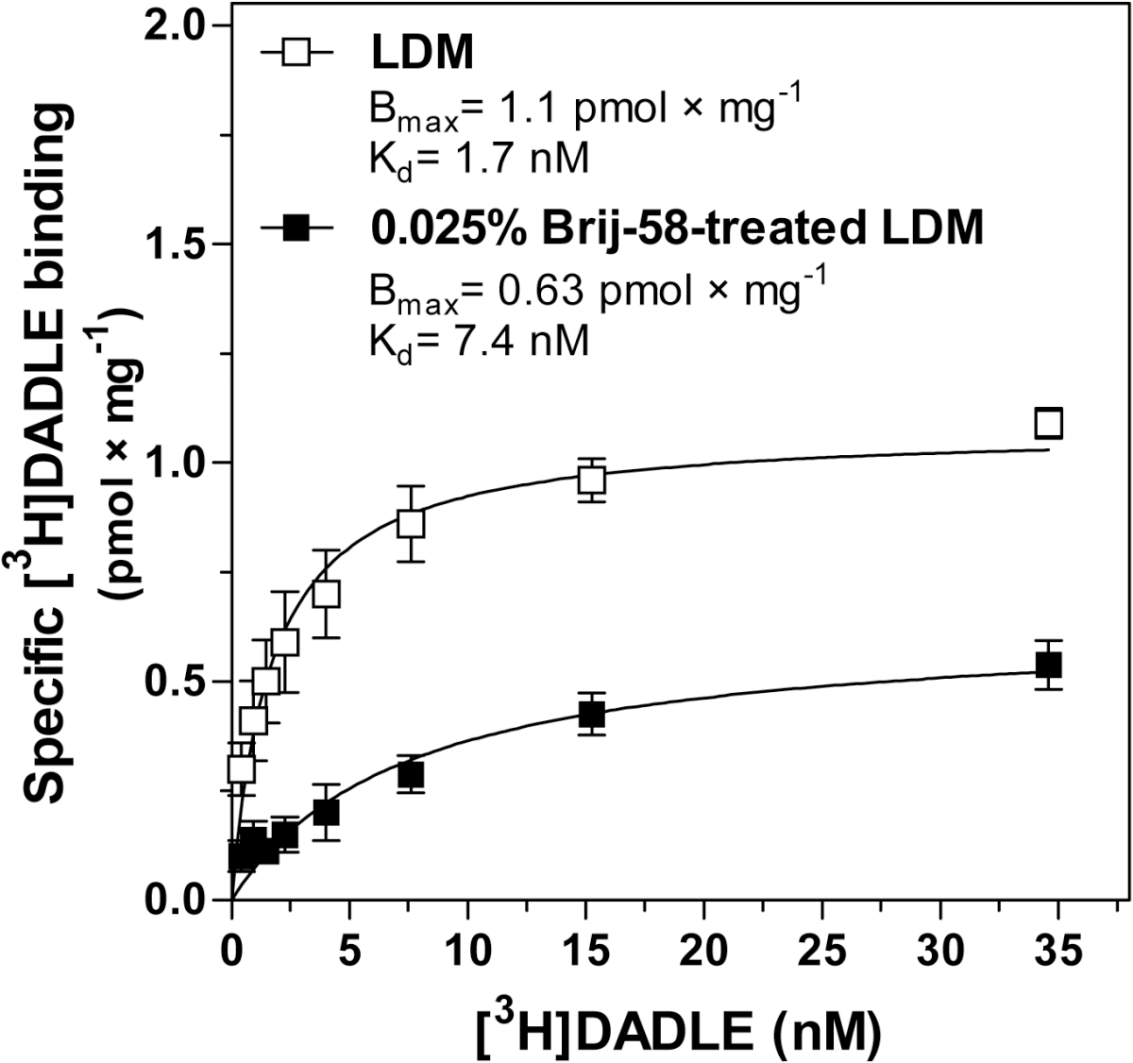
Saturation of [^3^H]DADLE binding sites in LDM and 0.025% Brij-58-treated LDM prepared from PTX-treated δ-OR-G_i_1α cells. Saturation of specific [^3^H]DADLE binding sites was measured as described in Methods. The B_max_ and K_d_ values were calculated by GraphPadPrism4. The data represent the average of 3 experiments ± SEM. LDM, fractions collected from gradient containing no detergent; 0.025% Brij-58-treated LDM, fractions collected from gradient containing 0.025% Brij-58

To obtain sufficient amount of protein for performance of a multiple assays, fractions 1–6 were combined together and used for determination of dose-response curves of DADLE-stimulated [^35^S]GTPγS binding (**Fig 10**) and saturation binding assays of δ-OR by [^3^H]DADLE (**Fig 11**). Comparison of the dose-response curves indicated that the potency of agonist response in 0.025% Brij-58-treated LDM (EC_50_ = 280 ± 15 nM) was significantly lower by one-order of magnitude than in LDM prepared in the absence of detergent (EC_50_ = 28 ± 4 nM) (**Fig 10A, S10 Table**). Contrarily, the % of agonist stimulation over the basal level in 0.025% Brij-58-treated LDM (372%) was higher than in LDM (283%) (**Fig 10B**).

Saturation of [^3^H]DADLE binding sites in combined fractions 1–6 indicated the significant decrease of both affinity and maximum number of δ-OR in 0.025% Brij-58-treated LDM (K_d_ = 7.4 ± 2.2 nM; B_max_ = 0.6 ± 0.1 pmol × mg^-1^) when compared with LDM prepared in the absence of detergent (K_d_ = 1.7 ± 0.7 nM; B_max_ = 1.1 ± 0.1 pmol × mg^-1^) (**Fig 11, S11 Table**).

Thus, determination of functional response of G proteins to agonist stimulation in membranes prepared from PTX-treated δ-OR-G_i_1α (C^351^I)-HEK293 cells and normalization of this response to δ-OR number determined by agonist binding assay confirmed our previous results obtained in analysis of membranes prepared from PTX-untreated δ-OR-G_i_1α (C^351^I)-HEK293 cells.

### Direct effect of Brij-58 on δ-OR-G protein coupling in isolated plasma membranes at 30°C

**Fig 12:**
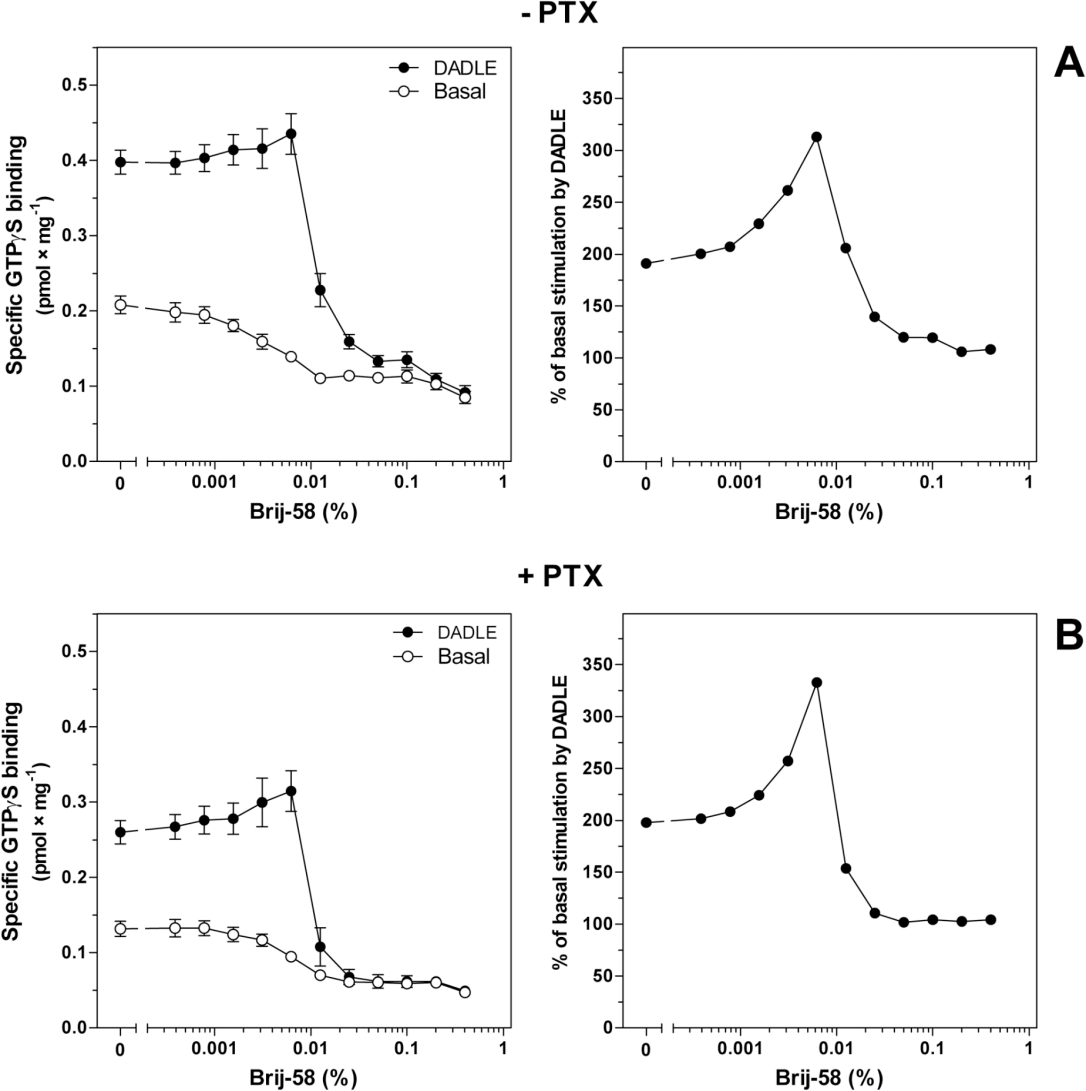
Direct effect of increasing concentrations of Brij-58 on basal and DADLE-stimulated [^35^S]GTPγS binding at 30°C; PM isolated from PTX-treated and untreated δ-OR-G_i_1α cells. Basal and DADLE-stimulated [^35^S]GTPγS binding was measured in Percoll-purified plasma membranes isolated from (**A**) PTX-untreated (-PTX) and (**B**) PTX-treated (+PTX) δ-OR-G_i_1α (C^351^I)-HEK293 cells. Increasing concentrations of Brij-58 were added directly into the assay medium and incubated for 30 min at 30°C. The data represent the average of 3 experiments ± SEM. Significance of difference between the specific DADLE-stimulated and basal [^35^S]GTPγS binding was determined by Student´s t-test (see S12 Table).

As noticed before in studies of baclofen-stimulated [^35^S]GTPγS binding in Percoll-purified PM prepared from rat brain cortex [29], the non-ionic detergents Brij-58, Triton X-100 and digitonin were able, within the narrow range of detergent concentrations, to increase GABA_B_-R agonist-stimulated [^35^S]GTPγS binding when added directly to the assay medium. Exposure to higher detergent concentrations resulted in drop of G protein activity to zero level. In the case of Brij-58, this range was between 0.006 and 0.013% (w/v). Similar result was observed in PM isolated from δ-OR-G_i_1α (C^351^I)-HEK293 cells: the increase of DADLE-stimulated [^35^S]GTPγS binding was detected at 0.006% Brij-58 (**Fig 12**). Importantly, activation of G proteins at this detergent concentration was detected in PM isolated from both PTX-untreated and PTX-treated δ-OR-G_i_1α cells (compare upper and lower panels in **Fig 12**). Therefore, the effect of Brij-58 on [^35^S]GTPγS binding was manifested for G_i_1α present within δ-OR-G_i_1α (C^351^I) fusion protein as well as for PTX-sensitive G proteins endogenously expressed in HEK293 cells.

**Fig 13:**
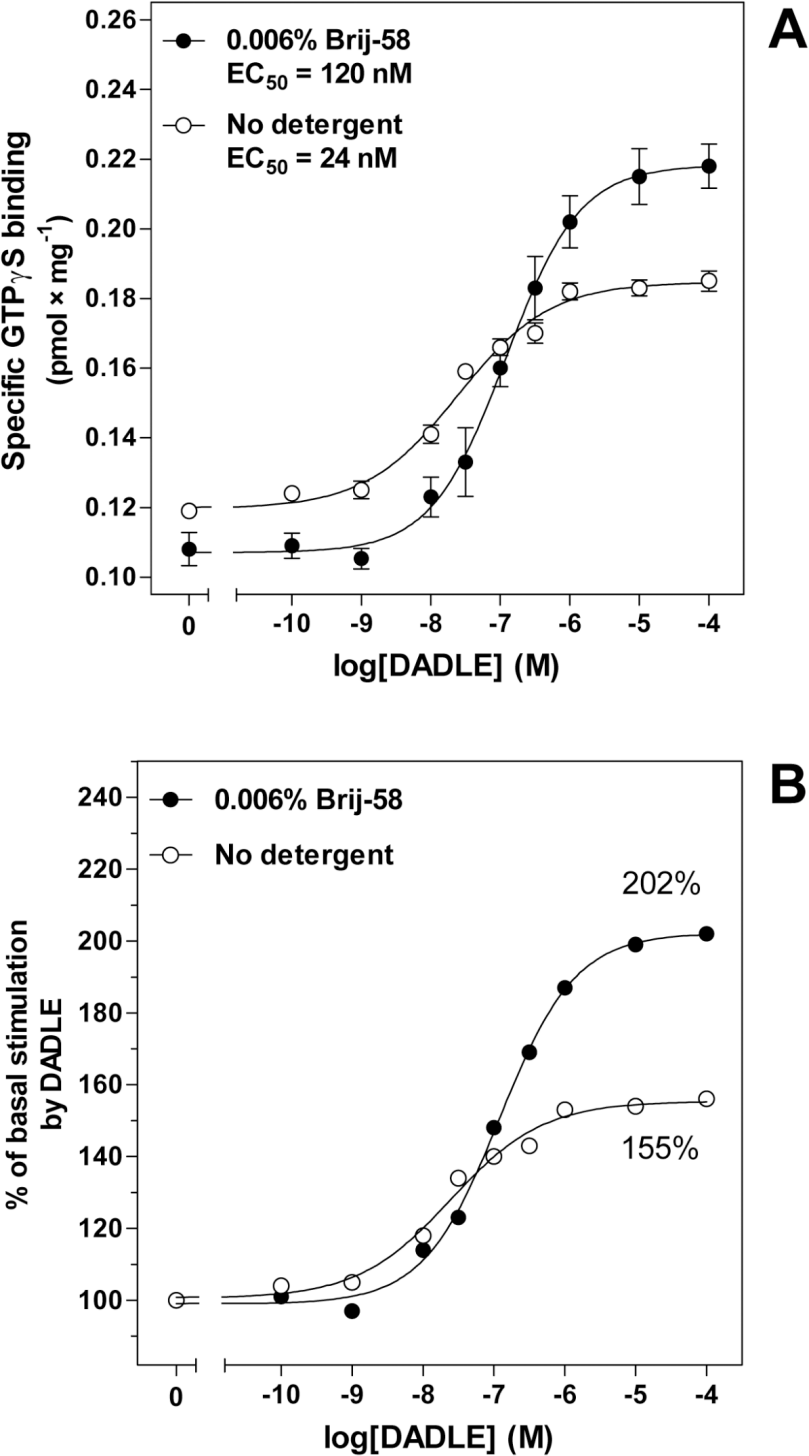
Dose-response curves of DADLE-stimulated [^35^S]GTPγS binding in PM isolated from δ-OR-G_i_1α cells; direct effect of 0.006% Brij-58 at 30°C. (**A**) Dose-response curves of DADLE-stimulated [^35^S]GTPγS binding were measured in plasma membranes isolated from δ-OR-G_i_1α (C^351^I)-HEK293 cells in the absence (○) or presence (●) of 0.006% Brij-58 as described in Methods in binding assay medium at 30°C. (**B**) The ratio between the DADLE-stimulated and the basal level of [^35^S]GTPγS binding was expressed as % of agonist stimulation over the basal level; the basal level represented 100%. The data represent the average of 3 experiments ± S.E.M.

**Fig 14:**
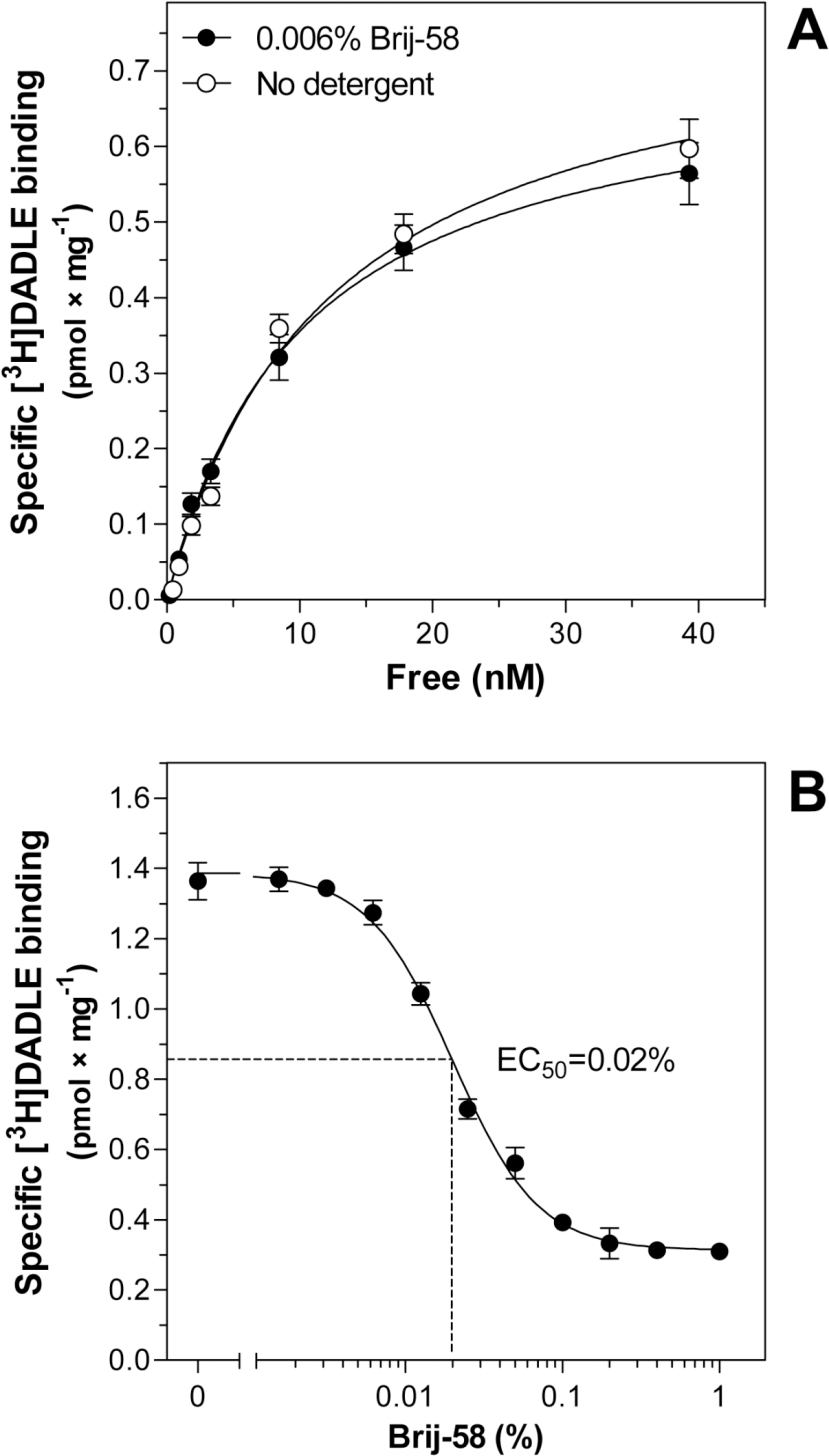
Saturation of [^3^H]DADLE binding sites in PM isolated from δ-OR-G_i_1α cells; direct effect of 0.006% Brij-58 at 30°C. (**A**) Saturation of δ-OR agonist [^3^H]DADLE binding sites was measured in plasma membranes isolated from δ-OR-G_i_1α (C^351^I)-HEK293 cells as described in Methods in the absence (○) or presence (●) of 0.006% Brij-58 in binding assay medium at 30°C. The B_max_ and K_d_ values were calculated by GraphPad Prism4. (**B**) Effect of increasing concentrations of Brij-58 on [^3^H]DADLE binding was measured in PM isolated in the absence of detergent; 50% inhibition of agonist binding was observed at 0.02% Brij-58. The data represent the average of 3 experiments ± S.E.M.

In accordance with the data obtained in analysis of LDM isolated at 0°C, comparison of the dose-response curves of DADLE-stimulated [^35^S]GTPγS binding in plasma membranes exposed to 0.006% Brij-58 at 30°C indicated a highly significant decrease in potency of agonist response: EC_50_ value was increased from 24 ± 5 nM (no detergent) to 120 ± 10 nM in PM exposed to 0.006% Brij-58 (**Fig 13, S13 Table**). The agonist binding to δ-OR was not affected at this detergent concentration (**Fig 14A, S14 Table**) and 0.02% concentration of Brij-58 was needed to achieve the half-maximum inhibition of [^3^H]DADLE binding (**Fig 14B**). It should be noticed that at this detergent concentration, DADLE-stimulated [^35^S]GTPγS binding was completely diminished (**Fig 12**). Therefore, agonist binding to δ-OR was substantially less sensitive to deteriorating effect of detergent than receptor ability to activate G proteins. This conclusion was in our experiments demonstrated for PTX-insensitive G_i_1α (C^351^I) covalently bound to δ-OR as well as for PTX-sensitive G proteins endogenously expressed in HEK293 cells.

### The effect of Brij-58 on hydrophobic plasma membrane interior

**Fig 15:**
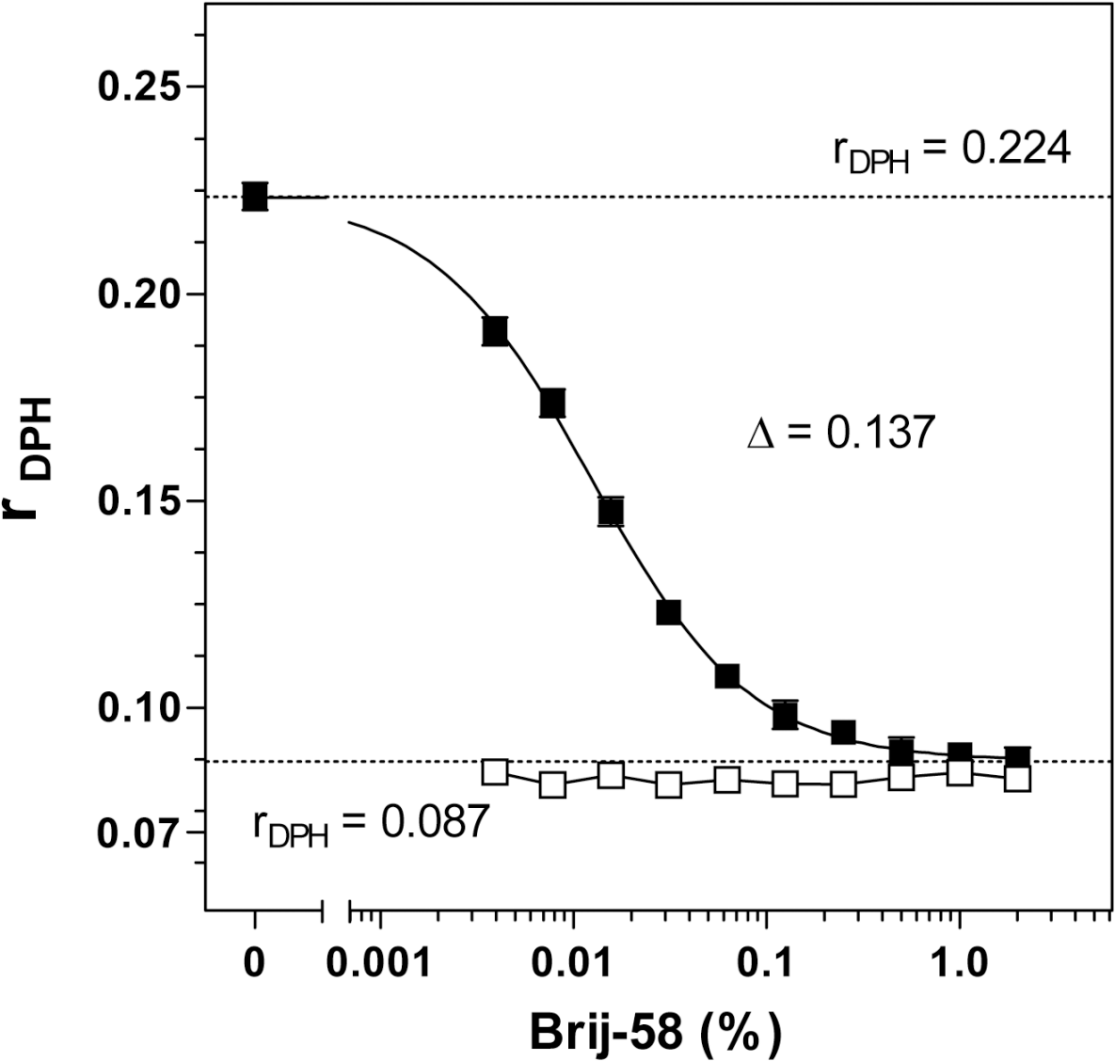
Effect of increasing concentrations of Brij-58 on „membrane fluidity”of isolated PM; steady-state anisotropy of fluorescence of diphenylhexatriene (r_DPH_). Suspension of Percoll-purified PM in 50 mM Tris-HCl, pH 7.4 (0.2 mg protein per ml) was quickly mixed with 1 mM DPH in acetone (to 1 μM final concentration) and incubated under stirring for 60 min at 25°C.Anisotropy of DPH fluorescence (r_DPH_) was measured at Ex 365/Em 425 nm wavelengths as described in Methods. (**■**), membranes plus Brij-58; (□), Brij-58 alone. The data shown represent the average of 3 experiments ± SEM.

Measurement of steady-state anisotropy of hydrophobic membrane probe DPH indicated that Brij-58 exhibited a strong “fluidization” of hydrophobic plasma membrane interior (**Fig 15**). When increasing Brij-58 above the critical micelle concentration (CMC = 0.0086% w/v), the highly polarized signal of DPH observed in detergent-untreated PM (r_DPH_ = 0.224 ± 0.003) was gradually decreased to the highly depolarized signal at high detergent concentrations (r_DPH_ = 0.087 ± 0.003), which was close to that of Brij-58 alone in aqueous medium at 25°C (r_DPH_ = 0.072 ± 0.001).

**Fig 16:**
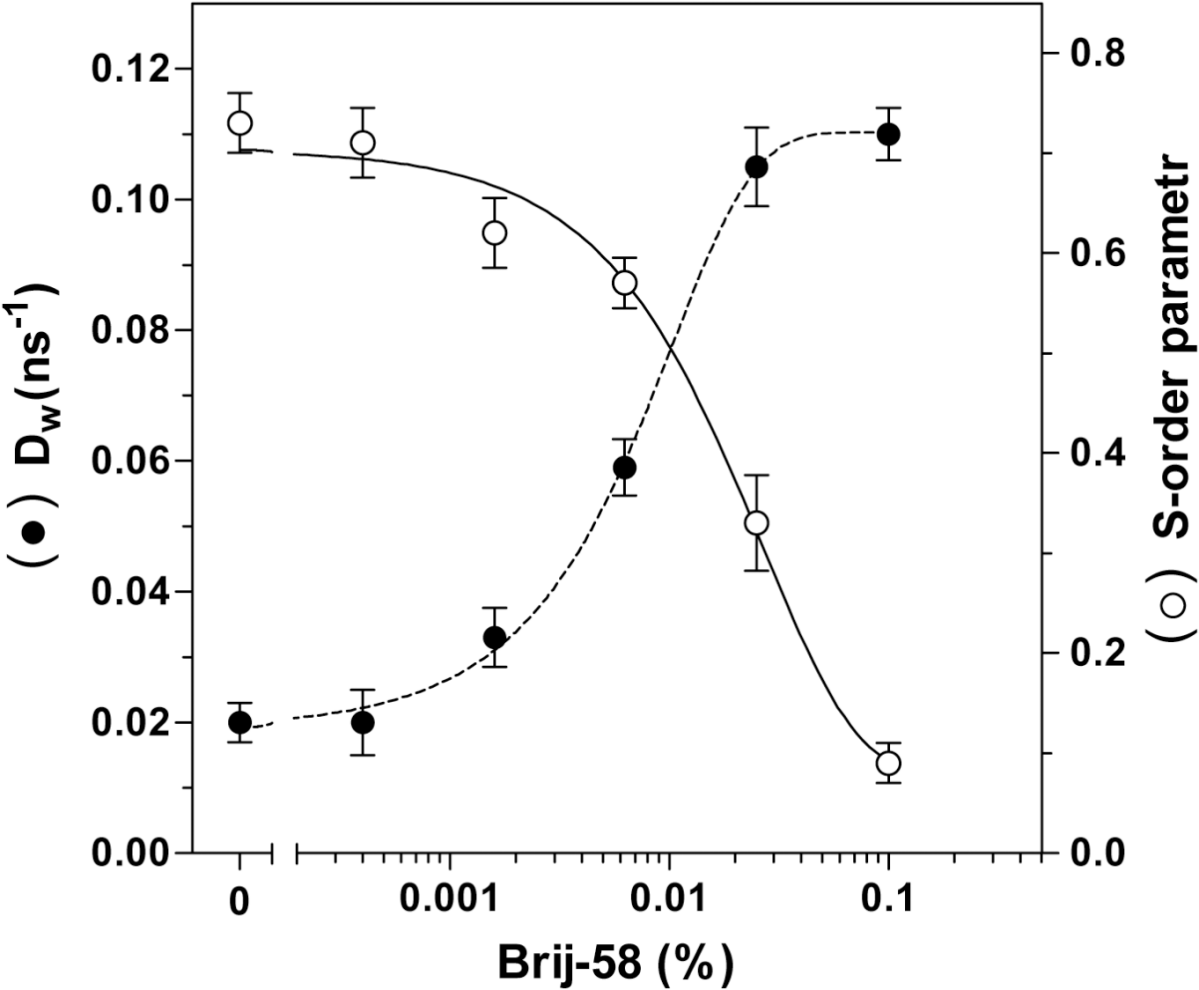
The effect of increasing concentrations of Brij-58 on time-resolved parameters of DPH fluorescence in PM; wobbling constant *D*_*w*_ and S-order parameter. The decay of anisotropy of DPH fluorescence in ns range was measured in PM isolated from PTX-untreated cells and analyzed as described in Methods. *D*_*w*_, wobbling diffusion constant (**●**) and S-order parameter (○) were calculated according to the “wobble in cone” model of DPH mobility in membrane bilayer. The data shown represent the average of three experiments.

More detailed understanding of the detergent effect on organization of PM microenvironment was obtained by determination of the lifetime values of DPH fluorescence and analysis of the time-resolved DPH anisotropy decay by “wobble in cone” model [30,31]. As shown in **Table 6**, the average lifetime of DPH fluorescence (*τ*_*av*_) was continuously decreased by increasing detergent concentrations. The S-order parameter decreased as well and this decrease proceeded in parallel with the increase of wobbling diffusion constant *D*_*w*_ (**Fig 16**). Accordingly, the values of rotational correlation time (*ϕ*) were decreased. Thus, the lower friction of DPH molecules within plasma membrane microenvironment and higher rate of DPH rotation was detected.

**Table 6:** The influence of increasing concentrations of Brij-58 on parameters of the time-resolved DPH fluorescence.

	*τ*_*1*_	*τ*_*2*_	*τ*_*av*_	*r*_*0*_	*r*_*∞*_	*ϕ*	*S*	*D*_*w*_
Brij-58 (%)								
**0**	2.68	9.70	8.35	0.26	0.136	5.59	0.73	0.020
**0.0004**	2.58	9.84	8.13	0.26	0.131	5.47	0.71	0.020
**0.0016**	2.29	9.50	7.68	0.27	0.103	4.26	0.62	0.033
**0.0062**	2.26	8.91	7.56	0.27	0.089	2.67	0.57	0.059
**0.025**	1.62	7.75	7.40	0.27	0.029	2.14	0.33	0.105
**0.1**	1.61	7.31	7.12	0.27	0.002	2.32	0.09	0.110

PM isolated from δ-OR-G_i_1α (C^351^I)-HEK293 cells were exposed to increasing concentrations of Brij-58 for 30 minutes at 25°C and labeled by DPH (1 μM, 30 min). Fluorescence lifetime and parameters of time-resolved decays of anisotropy of DPH fluorescence were determined as described in Methods. *τ*_*1*_ and *τ*_*2*_, the short- and long- components of lifetime of DPH fluorescence; *τ*_*av*_, average lifetime; *r*_*0*_, limiting anisotropy at time zero; ***r***_***∞***_, residual anisotropy; ***ϕ***, rotational correlation time; *S*, S-order parameter; *D*_*w*_, wobbling diffusion constant.

## Discussion

According to the collision-coupling model of receptor-G protein interaction [32], the level of G protein activated is independent of the concentration of agonist-bound receptors, but the rate of G protein activation is proportional to the concentration of agonist-bound receptors. Thus, reduction of receptor density should cause no reduction in the maximal level of G protein activation but it should reduce the rate of G protein activation and decrease the EC_50_ values of agonist-stimulated G protein activity. It was also suggested that diffusion of receptors and effectors is slow, so that receptors will only activate effectors that are in their vicinity at the time of agonist occupation [33,34]. The rate of G protein activation was found to be dependent on the agonist binding frequency relative to the encounter time of G protein and receptor [35,36].

The test of validity of collision-coupling model for opioid receptors was performed in C6 cells expressing different amounts of μ- and δ-OR and G proteins [37]. The time-course of full or partial agonist-stimulated [^35^S]GTPγS binding did not vary in cells containing widely different amounts of μ-OR or δ-OR and the 10-fold reduction of the functional complement of G proteins by pertussis toxin caused the decrease of maximum agonist-stimulated [^35^S]GTPγS binding. The association rate of [^35^S]GTPγS with agonist-activated G protein was unchanged. Results of this study were not compatible with the random collision-coupling model but supported a compartmentalization model [38–40] in which receptors and G proteins are “in some way” associated together and compartmentalized within plasma membrane.

The association of GPCRs and G proteins with the plasma membrane makes them susceptible to their lipid environment so that lipid-protein interactions are important for their function [41]. In direct relevance to theoretical models of receptor-G protein coupling, plasma membrane domains/rafts were suggested as devices concentrating the signaling molecules, i.e. bringing them into a closer spatial organization than just the random order arrangement within the fluid mosaic of proteins and lipids in the bulk of plasma membranes [1–6,9–12,16]. The content of trimeric G proteins and GPCR in these PM compartments responded dramatically to agonist stimulation [22,23,42–59].

More specifically, κ-OR were found to be localized in lipid rafts [60] and analysis of μ-OR- and δ-OR-G protein coupling indicated the compartmentalization of these signaling molecules in PM [37]. Cholesterol reduction by methyl-β-cyclodextrin was found to attenuate δ-OR-initiated signaling in neuronal cells but enhance it in non-neuronal cells [57]. In HEK293 cells stably expressing δ-OR-G_i_1α fusion protein, cholesterol depletion by β-cyclodextrin resulted in decrease of potency of G protein response to agonist DADLE stimulation, but the maximum response was unchanged [26]. Adenylyl cyclase super-activation induced by prolonged exposure to morphine was found to be dependent on receptor localized within lipid rafts and independent of receptor internalization [61]. Palmitoylation and membrane cholesterol stabilized the homo-oligomerization of μ-OR and coupling with G proteins [62].

Furthermore, experiments based on plasmon-waveguide resonance spectroscopy [63] indicated that the unbound, inactive δ-OR receptors were enriched in 1, 2-palmitoyl-oleoyl-sn-glycero-3-phosphoserine (POPC)-rich domain of lipid bilayer composed of 1:1 mixture of POPC and sphingomyelin (SM). The agonist- and antagonist-bound receptors were concentrated in SM-rich component (with a two-fold greater propensity in the case of the agonist) characterized by a greater thickness. Since it is known that receptor activation involves changes in the orientation of trans-membrane helices with an increase in receptor vertical length, i.e. perpendicular to the plane of the plasma membrane [64], it was hypothesized that the driving force for receptor redistribution comes from the increased hydrophobic match of the elongated receptor for the thicker SM-enriched part of the bilayer, a sorting mechanism that has previously been observed in other subcellular compartments [65,66].

Literature data mentioned above justify the effort to analyze in details the interaction of detergents with various plasma membrane preparations containing the functional GPCR and G proteins with the aim to find “some correlation” between the structural and dynamic state of the membrane lipid bilayer and parameters of GPCR-G protein coupling. Obviously, this type of analysis requires the step by step determination of concentration dependency of detergent effect.

Comparison of LDM isolated from PTX-untreated and PTX-treated δ-OR-G_i_1α (C^351^I)- HEK293 cells in the absence or presence of low concentrations of Brij-58 at 0°C indicated the decrease of δ-OR number in Brij-58-treated LDM when compared with detergent-untreated LDM (**Figs 7** and **9**). The decrease of δ-OR was measured by both agonist and antagonist binding assays and accompanied by a decrease in potency of agonist response (**Figs 4, 5** and **10**). This was demonstrated by comparison of the dose-response curves of both DADLE-stimulated [^32^P]GTPase (**Fig 4**) and [^35^S]GTPγS binding (**Figs 5** and **10**). However, the maximum G protein response (efficacy) was significantly increased (**Tables 1–5**). Similar data were obtained in analysis of the direct effect of increasing concentrations of Brij-58 on isolated plasma membranes at 30°C (**Figs 12–14**).

When interpreting these results, it is logical to assume that the increase of detergent concentration at hydrophobic interface of δ-OR (exposed to the bulk lipid phase of the membrane) removes lipids attached to the outer surface of protein molecule (lipid annulus) and in this way perturbs the optimum lipid environment of δ-OR needed for its optimum function. This is reflected first as a transitional increase of efficacy of G protein response to agonist stimulation accompanied by decrease of potency. Subsequently, at high detergent concentration, receptor is unable to find the proper, agonist-responding conformation and G protein response in completely diminished.

It may be also visualized that limited degradation of organized PM structure by detergent in critical range of low concentrations (0.01–0.1% Brij-58 at 0°C) might detach G proteins from the inhibitory effect of caveolin [15,50,67,68] or that the detergent-induced change of structural organization of lipid annulus surrounding δ-OR alters the oligomerization state of OR and, consequently, parameters of functional coupling between OR and their cognate G proteins [69–77].

Our previous results [29] indicated that DPH anisotropy (r_DPH_) represents a useful parameter for analysis of detergent effect on membranes isolated from brain. In δ-OR-G_i_1α (C^351^I)-HEK293 cells, the decrease of r_DPH_ by Brij-58 (**Fig 15**) was 5-fold higher than that induced by cholesterol depletion [26]. The highly polarized signal of DPH, measured in PM in absence of detergent (r_DPH_ = 0.224), was decreased to the highly depolarized signal (r_DPH_ = 0.087) detected at high detergent concentrations. The application of “wobble in cone” model enabled us to retrieve the information about membrane dynamics characterized by wobbling diffusion constant (*D*_*w*_). This model also enabled us to gain the “static” information about the degree of the orientation constrains of rotational movements of DPH dye by calculation of S-order parameter (*S*). The time-resolved analysis of DPH fluorescence by the “wobble in cone model” of DPH motion (**Table 6** and **Fig 16**) indicated that the exposure of PM to the increasing concentrations of Brij-58 led to a higher motional freedom of the dye (increase of *D*_*w*_) and a less structurally ordered structure of membrane bilayer (decrease of S-order parameter).

The existence of “critical range” of detergent concentrations inducing the limited membrane damage before the total collapse of plasma membrane structure and diminution of membrane protein function was also detected for GABA_B_-receptors in plasma membranes isolated from rat forebrain cortex [29]. In close analogy to the data presented in this work (**Fig 12**), the clearly defined stimulatory [Brij-58, 0.006–0.013% w/v; Triton X-100, 0.013–0.025%) and inhibitory [Brij-58, >0.02% w/v; Triton X-100, >0.05% w/v) range of detergent concentrations was observed.

## Conclusions

1) This work describes the simple method for preparation of intermediate forms of low-density, detergent-treated plasma membrane fragments (Brij-58-treated LDM) which are distinct from detergent-untreated PM fragments (LDM) exhibiting the same density but, on the other hand, do not represent the typical detergent-resistant membrane domains (DRMs) isolated in 0.5–1% Triton-X100 at 0°C. The “typical” DRMs contain less than 1% of original PM protein, large portion (30–40%) of trimeric G proteins, caveolin and flotilin but do not exhibit the functional coupling between δ-OR and G proteins due to deleterious effect of high detergent concentrations.

2) In optimum range of detergent concentrations (0.025–0.05% w/v), Brij-58-treated, low-density membranes exhibited 2-3-fold higher efficacy of DADLE-stimulated, high-affinity [γ-^32^P]GTPase and [^35^S]GTPγS binding than membranes of the same density prepared in the absence of detergent. The potency of agonist DADLE response was significantly decreased. At high detergent concentrations (>0.1%), the functional coupling between δ-OR and G proteins was completely diminished. The same detergent effects were measured in LDM isolated from PTX-treated cells. Therefore, the effect of Brij-58 on δ-OR-G protein coupling was not restricted to the covalently bound G_i_1α within δ-OR-G_i_1α fusion protein, but it was also valid for PTX-sensitive G proteins of G_i_/G_o_ family endogenously expressed in HEK293 cells.

## Supporting Information

S1 Table Statistical analysis of [^32^P]GTPase activity in gradient fractions. Sucrose density gradients (1)-(4) were prepared from PTX-untreated δ-OR-G_i_1α cells.
(DOCX)

S2 Table Statistical analysis of [^35^S]GTPγS binding in gradient fractions. Sucrose density gradients (1)-(3) were prepared from PTX-untreated δ-OR-G_i_1α cells.
(DOCX)

S3 Table Statistical analysis of [^32^P]GTPase activity in PNS, LDM and 0.025% Brij-58-treated LDM. (DOCX)

S4 Table Statistical analysis of dose-response curves of DADLE-stimulated [^32^P]GTPase activity. Comparison of PNS versus LDM and 0.025% Brij-58-treated LDM.
(DOCX)

S5 Table Statistical analysis of dose-response curves of DADLE-stimulated [^35^S]GTPγS binding. Comparison of PNS versus LDM and 0.025% Brij-58-treated LDM.
(DOCX)

S6 Table Statistical analysis of competitive [^35^S]GTPγS/GTPγS binding displacement curves. Comparison of PNS versus LDM and 0.025% Brij-58-treated LDM.
(DOCX)

S7 Table Statistical analysis of [^3^H]naltrindole saturation binding curves. Comparison of PNS versus LDM and 0.025% Brij-58-treated LDM.
(DOCX)

S8 Table Statistical analysis of [^35^S]GTPγS binding in gradient fractions. Sucrose density gradients were prepared from PTX-treated δ-OR-G_i_1α cells in absence (no detergent) or presence of 0.025% Brij-58.
(DOCX)

S9 Table Statistical analysis of [^3^H]DADLE binding in gradient fractions. Sucrose density gradients were prepared from PTX-treated δ-OR-G_i_1α cells.
(DOCX)

S10 Table Statistical analysis of dose-response curves of DADLE-stimulated [^35^S]GTPγS binding. LDM versus 0.025% Brij-58-treated LDM prepared from PTX-treated δ-OR-G_i_1α cells.
(DOCX)

S11 Table Statistical analysis of [^3^H]DADLE saturation binding curves. LDM versus 0.025% Brij-58-treated LDM prepared from PTX-treated δ-OR-G_i_1α cells.
(DOCX)

S12 Table Statistical analysis of [^35^S]GTPγS binding. Direct effect of increasing concentrations of Brij-58 in PM isolated from PTX-untreated and PTX-treated δ-OR-G_i_1α cells.
(DOCX)

S13 Table Statistical analysis of dose-response curves of DADLE-stimulated [^35^S]GTPγS binding. Direct effect of 0.006% Brij-58 in PM.
(DOCX)

S14 Table Statistical analysis of [^3^H]DADLE saturation binding curves. Direct effect of 0.006% Brij-58 in PM.
(DOCX)
