# Supplementary material for: High Efficacy but Low Potency of δ-Opioid Receptor-G Protein Coupling in Brij-58-Treated, Low-Density Plasma Membrane Fragments
Source: PLoS One. 2015 Aug 18;10(8):e0135664. doi: 10.1371/journal.pone.0135664 (PMC4540457; doi:10.1371/journal.pone.0135664)
Supplement: S1 Table — Sucrose density gradients (1)-(4) were prepared from PTX-untreated δ-OR-Gi1α cells. (DOCX) [file pone.0135664.s001.docx]

**S1 Table. Statistical analysis of [^32^P]GTPase activity in gradient fractions.**

Sucrose density gradients (1)-(4) were prepared from *PTX-untreated δ-OR-G_i_1α* cells.

| **(A) *Student´s t-test* Basal** vs. **DADLE-stimulated GTPase activity** | | | | | | | | |
| --- | --- | --- | --- | --- | --- | --- | --- | --- |
| **gradient** | **(1) No detergent** | | **(2) 0.025% Brij-58** | | **(3) 0.05% Brij-58** | | **(4) 0.1% Brij-58** | |
| **Fraction** | **P value** | **P value summary** | **P value** | **P value summary** | **P value** | **P value summary** | **P value** | **P value summary** |
| **1** | p>0.05 | ND | p<0.05 | * | p<0.001 | *** | p>0.05 | ND |
| **2** | p<0.05 | * | p<0.001 | *** | p<0.001 | *** | p>0.05 | ND |
| **3** | p<0.01 | ** | p<0.001 | *** | p<0.001 | *** | p>0.05 | ND |
| **4** | p<0.001 | *** | p<0.001 | *** | p<0.001 | *** | p>0.05 | ND |
| **5** | p<0.001 | *** | p<0.001 | *** | p<0.05 | * | p>0.05 | ND |
| **6** | p<0.01 | ** | p<0.01 | ** | p>0.05 | ND | p>0.05 | ND |
| **7** | p<0.05 | * | p<0.01 | ** | p>0.05 | ND | p>0.05 | ND |
| **8** | p>0.05 | ND | p>0.05 | ND | p>0.05 | ND | p>0.05 | ND |
| **9** | p>0.05 | ND | p>0.05 | ND | p>0.05 | ND | p>0.05 | ND |
| **10** | p>0.05 | ND | p>0.05 | ND | p>0.05 | ND | p>0.05 | ND |
| **11** | p>0.05 | ND | p>0.05 | ND | p>0.05 | ND | p>0.05 | ND |
| **12** | p>0.05 | ND | p>0.05 | ND | p>0.05 | ND | p>0.05 | ND |

| **(B) *One-way ANOVA* No detergent** vs. **0.025% Brij-58** vs. **0.05% Brij-58** vs. **0.1% Brij-58** | | | |
| --- | --- | --- | --- |
| Parameter | | **Δ_DADLE_** | |
|  | P value | p<0.001 | |
|  | P value summary | *** | |
|  | Are means signif. different? | Yes | |
|  | **Bonferroni's Multiple Comparison Test** | | |
|  |  | **Significant? (**p<0.05) | **P value summary** |
|  | **No detergent** vs. **0.025% Brij-58** | Yes | ** |
|  | **No detergent** vs. **0.05% Brij-58** | Yes | ** |
|  | **No detergent** vs. **0.1% Brij-58** | Yes | ** |
|  | **0.025% Brij-58** vs. **0.05% Brij-58** | No | ND |
|  | **0.025% Brij-58** vs. **0.1% Brij-58** | Yes | *** |
|  | **0.05% Brij-58** vs. **0.1% Brij-58** | Yes | *** |

**(A)** The significance of difference between the specific DADLE-stimulated and basal [^32^P]GTPase activity (Fig. 1) in fractions 1-12 collected from sucrose density gradients was determined by Student´s t-test

**(B)** Net increment of agonist stimulation (Δ_DADLE_) was calculated as the difference between specific DADLE-stimulated and basal [^32^P]GTPase activity (pmol × min^-1^ × mg^-1^ protein) in fractions 1-6. The significance of difference of Δ_DADLE_ values in sucrose density gradients was determined by one-way ANOVA followed by Bonferroni´s multiple comparison test

* (p<0.05), significant difference; ** (p<0.01), *** (p<0.001), highly significant difference; ND (p>0.05), not different
