# Supplementary material for: High Efficacy but Low Potency of δ-Opioid Receptor-G Protein Coupling in Brij-58-Treated, Low-Density Plasma Membrane Fragments
Source: PLoS One. 2015 Aug 18;10(8):e0135664. doi: 10.1371/journal.pone.0135664 (PMC4540457; doi:10.1371/journal.pone.0135664)
Supplement: S3 Table — (DOCX) [file pone.0135664.s003.docx]

**S3 Table. Statistical analysis of [^32^P]GTPase activity in PNS, LDM and 0.025% Brij-58-treated LDM.**

| **(A)**  ***Student´s t-test*** | | **Basal** vs. **DADLE-stimulated GTPase activity** | | | |
| --- | --- | --- | --- | --- | --- |
|  | Parameter | **V_max_** | | **K_m_** | |
|  |  | **P value** | **P value summary** | **P value** | **P value summary** |
|  | **PNS** | p>0.05 | ND | p>0.05 | ND |
|  | **LDM** | p<0.001 | *** | p>0.05 | ND |
|  | **0.025% Brij-58-treated LDM** | p<0.001 | *** | p>0.05 | ND |

| **(B) *One-way ANOVA*** | | **PNS** vs. **LDM** vs. **0.025%** **Brij-58-LDM** | | | |
| --- | --- | --- | --- | --- | --- |
| **Basal GTPase activity** | | **V_max_** | | **K_m_** | |
|  | P value | p<0.01 | | p<0.05 | |
|  | P value summary | ** | | * | |
|  | Are means signif. different? | Yes | | Yes | |
|  | **Bonferroni's Multiple Comparison Test** | | | | |
|  |  | **V_max_** | | **K_m_** | |
|  |  | **Significant? (**p<0.05) | **P value summary** | **Significant? (**p<0.05) | **P value summary** |
|  | **PNS** vs. **LDM** | Yes | * | No | ND |
|  | **PNS** vs. **0.025%** **Brij-58-LDM** | No | ND | Yes | * |
|  | **LDM** vs. **0.025%** **Brij-58-LDM** | Yes | * | No | ND |
| **DADLE*-*stimulated GTPase activity** | | **V_max_** | | **K_m_** | |
|  | P value | p<0.001 | | p<0.05 | |
|  | P value summary | *** | | * | |
|  | Are means signif. different? | Yes | | Yes | |
|  | **Bonferroni's Multiple Comparison Test** | | | | |
|  |  | **V_max_** | | **K_m_** | |
|  |  | **Significant? (**p<0.05) | **P value summary** | **Significant? (**p<0.05) | **P value summary** |
|  | **PNS** vs. **LDM** | Yes | *** | No | ND |
|  | **PNS** vs. **0.025%** **Brij-58-LDM** | Yes | *** | Yes | * |
|  | **LDM** vs. **0.025%** **Brij-58-LDM** | No | ND | No | ND |

Post-nuclear supernatant (PNS), low-density fractions (LDM) and 0.025% Brij-58-treated LDM were prepared from δ-OR-G_i_1α cells (Fig. 3)

**(A)** The significance of difference of V_max_ and K_m_ parameters between the basal and DADLE-stimulated [^32^P]GTPase activity was determined by Student´s t-test

**(B)** Comparison of V_max_ and K_m_ in PNS versus LDM versus 0.025% Brij-58-treated LDM was performed by one-way ANOVA followed by Bonferroni´s multiple comparison test

* (p<0.05), significant difference; ** (p<0.01), *** (p<0.001), highly significant difference; ND (p>0.05), not different
