# Supplementary material for: High Efficacy but Low Potency of δ-Opioid Receptor-G Protein Coupling in Brij-58-Treated, Low-Density Plasma Membrane Fragments
Source: PLoS One. 2015 Aug 18;10(8):e0135664. doi: 10.1371/journal.pone.0135664 (PMC4540457; doi:10.1371/journal.pone.0135664)
Supplement: S4 Table — Comparison of PNS versus LDM and 0.025% Brij-58-treated LDM. (DOCX) [file pone.0135664.s004.docx]

**S4 Table.** **Statistical analysis of dose-response curves of DADLE-stimulated [^32^P]GTPase activity.**

Comparison of PNS versus LDM and 0.025% Brij-58-treated LDM.

| ***One-way ANOVA*** | | **PNS** vs. **LDM** vs. **0.025%** **Brij-58-LDM** | | | |
| --- | --- | --- | --- | --- | --- |
| Parameter | | **EC_50_** | | **Δ_DADLE_** | |
|  | P value | p<0.001 | | p<0.01 | |
|  | P value summary | *** | | ** | |
|  | Are means signif. different? | Yes | | Yes | |
|  | **Bonferroni's Multiple Comparison Test** | | | | |
|  |  | **EC_50_** | | **Δ_DADLE_** | |
|  |  | **Significant? (**p<0.05) | **P value summary** | **Significant? (**p<0.05) | **P value summary** |
|  | **PNS** vs. **LDM** | No | ND | Yes | * |
|  | **PNS** vs. **0.025%** **Brij-58-LDM** | Yes | ******* | Yes | ** |
|  | **LDM** vs. **0.025%** **Brij-58-LDM** | Yes | ******* | Yes | * |

The significance of difference of EC_50_ and Δ_DADLE_ parameters (Fig. 4) was determined by one-way ANOVA followed by Bonferroni´s multiple comparison test

* (p<0.05), significant difference; ** (p<0.01), *** (p<0.001), highly significant difference; ND (p>0.05), not different
