# Supplementary material for: High Efficacy but Low Potency of δ-Opioid Receptor-G Protein Coupling in Brij-58-Treated, Low-Density Plasma Membrane Fragments
Source: PLoS One. 2015 Aug 18;10(8):e0135664. doi: 10.1371/journal.pone.0135664 (PMC4540457; doi:10.1371/journal.pone.0135664)
Supplement: S6 Table — Comparison of PNS versus LDM and 0.025% Brij-58-treated LDM. (DOCX) [file pone.0135664.s006.docx]

**S6 Table.** **Statistical analysis of competitive [^35^S]GTPγS/GTPγS binding displacement curves**.

Comparison of PNS versus LDM and 0.025% Brij-58-treated LDM.

| ***One-way ANOVA* PNS** vs. **LDM** vs. **0.025%** **Brij-58-LDM** | | | |
| --- | --- | --- | --- |
| Parameter | | **IC_50_** | |
| **-DADLE** | P value | p>0.05 | |
|  | P value summary | ND | |
|  | Are means signif. different? | No | |
|  | **Bonferroni's Multiple Comparison Test** | | |
|  |  | **Significant? (**p<0.05) | **P value summary** |
|  | **PNS** vs. **LDM** | No | ND |
|  | **PNS** vs. **0.025%** **Brij-58-LDM** | No | ND |
|  | **LDM** vs. **0.025%** **Brij-58-LDM** | No | ND |
|  | | **IC_50_** | |
| **+DADLE** | P value | p>0.05 | |
|  | P value summary | ND | |
|  | Are means signif. different? | No | |
|  | **Bonferroni's Multiple Comparison Test** | | |
|  |  | **Significant? (**p<0.05) | **P value summary** |
|  | **PNS** vs. **LDM** | No | ND |
|  | **PNS** vs. **0.025%** **Brij-58-LDM** | No | ND |
|  | **LDM** vs. **0.025%** **Brij-58-LDM** | No | ND |
|  | | | |
| Parameter | | **ΔB_0_** | |
|  | P value | p<0.001 | |
|  | P value summary | *** | |
|  | Are means signif. different? | Yes | |
|  | **Bonferroni's Multiple Comparison Test** | | |
|  |  | **Significant? (**p<0.05) | **P value summary** |
|  | **PNS** vs. **LDM** | Yes | ** |
|  | **PNS** vs. **0.025%** **Brij-58-LDM** | Yes | *** |
|  | **LDM** vs. **0.025%** **Brij-58-LDM** | Yes | ** |

The significance of difference of IC_50_ and ΔB_0_ parameters (Fig. 6 was determined by one-way ANOVA followed by Bonferroni´s multiple comparison test

* (p<0.05), significant difference; ** (p<0.01), *** (p<0.001), highly significant difference; ND (p>0.05), not different
