# Supplementary material for: High Efficacy but Low Potency of δ-Opioid Receptor-G Protein Coupling in Brij-58-Treated, Low-Density Plasma Membrane Fragments
Source: PLoS One. 2015 Aug 18;10(8):e0135664. doi: 10.1371/journal.pone.0135664 (PMC4540457; doi:10.1371/journal.pone.0135664)
Supplement: S8 Table — Sucrose density gradients were prepared from PTX-treated δ-OR-Gi1α cells in absence (no detergent) or presence of 0.025% Brij-58. (DOCX) [file pone.0135664.s008.docx]

**S8 Table. Statistical analysis of [^35^S]GTPγS binding in gradient fractions.**

Sucrose density gradients were prepared from *PTX-treated δ-OR-G_i_1α* cells in absence (no detergent) or presence of 0.025% Brij-58.

| ***Student´s t-test* Basal** vs. **DADLE-stimulated GTPγS binding** | | | | |
| --- | --- | --- | --- | --- |
| **gradient** | **No detergent** | | **0.025% Brij-58** | |
| **Fraction** | **P value** | **P value summary** | **P value** | **P value summary** |
| **1** | p>0.05 | ND | p>0.05 | ND |
| **2** | p>0.05 | ND | p<0.01 | ** |
| **3** | p<0.05 | * | p<0.01 | ** |
| **4** | p<0.05 | * | p<0.001 | *** |
| **5** | p<0.001 | *** | p<0.001 | *** |
| **6** | p<0.001 | *** | p<0.001 | *** |
| **7** | p<0.01 | ** | p<0.01 | ** |
| **8** | p>0.05 | ND | p>0.05 | ND |
| **9** | p>0.05 | ND | p>0.05 | ND |
| **10** | p>0.05 | ND | p>0.05 | ND |
| **11** | p>0.05 | ND | p>0.05 | ND |

The significance of difference between the specific DADLE-stimulated and basal [^35^S]GTPγS binding (Fig. 8) was determined by Student´s t-test

* (p<0.05), significant difference; ** (p<0.01), *** (p<0.001), highly significant difference; ND (p>0.05), not different
