# Supplementary material for: High Efficacy but Low Potency of δ-Opioid Receptor-G Protein Coupling in Brij-58-Treated, Low-Density Plasma Membrane Fragments
Source: PLoS One. 2015 Aug 18;10(8):e0135664. doi: 10.1371/journal.pone.0135664 (PMC4540457; doi:10.1371/journal.pone.0135664)
Supplement: S9 Table — Sucrose density gradients were prepared from PTX-treated δ-OR-Gi1α cells. (DOCX) [file pone.0135664.s009.docx]

**S9 Table. Statistical analysis of [^3^H]DADLE binding in gradient fractions.**

Sucrose density gradients were prepared from *PTX-treated δ-OR-G_i_1α* cells.

| ***Student´s t-test* No detergent** vs. **0.025% Brij-58** | | |
| --- | --- | --- |
| **Fraction** | **P value** | **P value summary** |
| **1** | p>0.05 | ND |
| **2** | p>0.05 | ND |
| **3** | p>0.05 | ND |
| **4** | p<0.001 | *** |
| **5** | p<0.001 | *** |
| **6** | p<0.001 | *** |
| **7** | p<0.01 | ** |
| **8** | p>0.05 | ND |
| **9** | p>0.05 | ND |
| **10** | p>0.05 | ND |
| **11** | p>0.05 | ND |

The significance of difference of [^3^H]DADLE binding (Fig. 9) in fractions prepared in absence (no detergent) or presence of 0.025% Brij-58 was determined by Student´s t-test

* (p<0.05), significant difference; ** (p<0.01), *** (p<0.001), highly significant difference; ND (p>0.05), not different
