# Supplementary material for: High Efficacy but Low Potency of δ-Opioid Receptor-G Protein Coupling in Brij-58-Treated, Low-Density Plasma Membrane Fragments
Source: PLoS One. 2015 Aug 18;10(8):e0135664. doi: 10.1371/journal.pone.0135664 (PMC4540457; doi:10.1371/journal.pone.0135664)
Supplement: S10 Table — LDM versus 0.025% Brij-58-treated LDM prepared from PTX-treated δ-OR-Gi1α cells. (DOCX) [file pone.0135664.s010.docx]

**S10 Table. Statistical analysis of dose-response curves of DADLE-stimulated [^35^S]GTPγS binding.**

LDM versus 0.025% Brij-58-treated LDM prepared from *PTX-treated δ-OR-G_i_1α* cells.

| ***Student´s t-test*** | **LDM** vs. **0.025%** **Brij-58-LDM** | |
| --- | --- | --- |
| Parameter | **EC_50_** | |
|  | **P value** | **P value summary** |
|  | p<0.001 | *** |

The significance of difference of EC_50_ parameter (Fig. 10) was determined by Student´s t-test

* (p<0.05), significant difference; ** (p<0.01), *** (p<0.001), highly significant difference; ND (p>0.05), not different
