# Supplementary material for: High Efficacy but Low Potency of δ-Opioid Receptor-G Protein Coupling in Brij-58-Treated, Low-Density Plasma Membrane Fragments
Source: PLoS One. 2015 Aug 18;10(8):e0135664. doi: 10.1371/journal.pone.0135664 (PMC4540457; doi:10.1371/journal.pone.0135664)
Supplement: S12 Table — Direct effect of increasing concentrations of Brij-58 in PM isolated from PTX-untreated and PTX-treated δ-OR-Gi1α cells. (DOCX) [file pone.0135664.s012.docx]

**S12 Table. Statistical analysis of [^35^S]GTPγS binding.**

Direct effect of increasing concentrations of Brij-58 in PM isolated from *PTX-untreated* and *PTX-treated δ-OR-G_i_1α* cells.

| ***Student´s t-test*** | **Basal** vs. **DADLE-stimulated GTPγS binding** | | | |
| --- | --- | --- | --- | --- |
|  | **-PTX** | | **+PTX** | |
| **Brij-58 (%)** | **P value** | **P value summary** | **P value** | **P value summary** |
| 0.0 | p<0.001 | *** | p<0.001 | *** |
| 0.00039 | p<0.001 | *** | p<0.001 | *** |
| 0.00078 | p<0.001 | *** | p<0.001 | *** |
| 0.00156 | p<0.001 | *** | p<0.001 | *** |
| 0.00313 | p<0.001 | *** | p<0.001 | *** |
| 0.00625 | p<0.001 | *** | p<0.001 | *** |
| 0.0125 | p<0.01 | ** | p<0.05 | * |
| 0.025 | p<0.05 | * | p>0.05 | ND |
| 0.05 | p>0.05 | ND | p>0.05 | ND |
| 0.1 | p>0.05 | ND | p>0.05 | ND |
| 0.2 | p>0.05 | ND | p>0.05 | ND |
| 0.4 | p>0.05 | ND | p>0.05 | ND |

The significance of difference between basal and DADLE-stimulated [^35^S]GTPγS binding (Fig. 12) was determined by Student´s t-test

* (p<0.05), significant difference; ** (p<0.01), *** (p<0.001), highly significant difference; ND (p>0.05), not different
