# Supplementary material for: High Efficacy but Low Potency of δ-Opioid Receptor-G Protein Coupling in Brij-58-Treated, Low-Density Plasma Membrane Fragments
Source: PLoS One. 2015 Aug 18;10(8):e0135664. doi: 10.1371/journal.pone.0135664 (PMC4540457; doi:10.1371/journal.pone.0135664)
Supplement: S14 Table — Direct effect of 0.006% Brij-58 in PM. (DOCX) [file pone.0135664.s014.docx]

**S14 Table. Statistical analysis of [^3^H]DADLE saturation binding curves.**

Direct effect of 0.006% Brij-58 in PM.

| ***Student´s t-test*** | **No detergent** vs. **0.006% Brij-58** | | | |
| --- | --- | --- | --- | --- |
| Parameter | **B_max_** | | **K_d_** | |
|  | **P value** | **P value summary** | **P value** | **P value summary** |
|  | p>0.05 | ND | p>0.05 | ND |

The significance of difference of B_max_ and K_d_ parameters (Fig. 14A) in PM isolated in absence (no detergent) or presence of 0.006% Brij-58 was determined by Student´s t-test

* (p<0.05), significant difference; ** (p<0.01), *** (p<0.001), highly significant difference; ND (p>0.05), not different
